# Peptidoglycan editing in non-proliferating intracellular *Salmonella* as source of interference with immune signaling

**DOI:** 10.1371/journal.ppat.1010241

**Published:** 2022-01-25

**Authors:** Sara B. Hernández, Sónia Castanheira, M. Graciela Pucciarelli, Juan J. Cestero, Gadea Rico-Pérez, Alberto Paradela, Juan A. Ayala, Sonsoles Velázquez, Ana San-Félix, Felipe Cava, Francisco García-del Portillo

**Affiliations:** 1 Laboratory for Molecular Infection Medicine Sweden, Department of Molecular Biology, Umeå Centre for Microbial Research, Umeå University, Umeå, Sweden; 2 Laboratory of Intracellular Bacterial Pathogens, National Centre for Biotechnology (CNB)-CSIC, Madrid, Spain; 3 Department of Molecular Biology, Universidad Autónoma de Madrid (UAM), Madrid, Spain; 4 Center for Molecular Biology “Severo Ochoa” (CBMSO)-CSIC, Madrid, Spain; 5 Proteomics Unit, National Centre for Biotechnology (CNB)-CSIC, Madrid, Spain; 6 Institute of Medicinal Chemistry (IQM)-CSIC, Madrid, Spain; University of California Davis School of Medicine, UNITED STATES

## Abstract

*Salmonella enterica* causes intracellular infections that can be limited to the intestine or spread to deeper tissues. In most cases, intracellular bacteria show moderate growth. How these bacteria face host defenses that recognize peptidoglycan, is poorly understood. Here, we report a high-resolution structural analysis of the minute amounts of peptidoglycan purified from *S*. *enterica* serovar Typhimurium (*S*. Typhimurium) infecting fibroblasts, a cell type in which this pathogen undergoes moderate growth and persists for days intracellularly. The peptidoglycan of these non-proliferating bacteria contains atypical crosslinked muropeptides with stem peptides trimmed at the L-alanine-D-glutamic acid-(γ) or D-glutamic acid-(γ)-*meso*-diaminopimelic acid motifs, both sensed by intracellular immune receptors. This peptidoglycan has a reduced glycan chain average length and ~30% increase in the L,D-crosslink, a type of bridge shared by all the atypical crosslinked muropeptides identified. The L,D-transpeptidases LdtD (YcbB) and LdtE (YnhG) are responsible for the formation of these L,D-bridges in the peptidoglycan of intracellular bacteria. We also identified in a fraction of muropeptides an unprecedented modification in the peptidoglycan of intracellular *S*. Typhimurium consisting of the amino alcohol alaninol replacing the terminal (fourth) D-alanine. Alaninol was still detectable in the peptidoglycan of a double mutant lacking LdtD and LdtE, thereby ruling out the contribution of these enzymes to this chemical modification. Remarkably, all multiple mutants tested lacking candidate enzymes that either trim stem peptides or form the L,D-bridges retain the capacity to modify the terminal D-alanine to alaninol and all attenuate NF-κB nuclear translocation. These data inferred a potential role of alaninol-containing muropeptides in attenuating pro-inflammatory signaling, which was confirmed with a synthetic tetrapeptide bearing such amino alcohol. We suggest that the modification of D-alanine to alaninol in the peptidoglycan of non-proliferating intracellular *S*. Typhimurium is an editing process exploited by this pathogen to evade immune recognition inside host cells.

## Author summary

The peptidoglycan, built as a giant polymer of glycan chains crosslinked with short peptides, is essential for cell shape and survival in most bacteria. Its unique chemistry is recognized by innate immune receptors, thereby enabling neutralization of invading microbes. A striking feature of the peptidoglycan is its constant remodeling by a plethora of endogenous enzymes. In addition, some bacterial pathogens introduce structural modifications that interfere with immune recognition. These modifications have been characterized in pathogens mostly in laboratory nutrient media. Whether facultative intracellular pathogens modify peptidoglycan structure inside host cells, was unknown. The work presented here shows that non-proliferating *Salmonella enterica* serovar Typhimurium remodels the peptidoglycan structure in response to intracellular cues and that some of these modifications involve unprecedented changes as the presence of an amino alcohol that hampers activation of the master immune regulator NF-κB. Peptidoglycan editing might therefore empower persistence of bacterial pathogens in the intracellular niche.

## Introduction

*Salmonella enterica* serovar Typhimurium (*S*. Typhimurium) is an important bacterial pathogen causing self-limiting gastroenteritis in humans and livestock [1] as well as systemic infections in mice and immunocompromised hosts [2]. *S*. Typhimurium has been widely studied in tissue culture and in animal infection models with regard to its intracellular lifestyle [3,4]. One of its most remarkable features is the diversity of physiological states that intracellular bacteria exhibit *in vivo* in target organs like the spleen and liver, with predominance of slow growing and non-proliferating populations [5,6]. Intracellular bacterial proliferation is also attenuated by the pathogen within non-phagocytic cells located in the intestinal lamina propria [7]. These physiological stages are understood as balanced attack and counter-attack strategies used by the host and pathogen. The host reacts to intracellular *S*. Typhimurium with defenses involving autophagy [8], exposure to lysosome content [9], oxidative/nitrosative bursts [10–12] and nutritional limitation [13]; whereas the pathogen responds by modeling a phagosome that hinders discharge of toxic compounds [14], avoids destruction by autophagy [15,16] and gains access to nutrients via an intricate network of tubules [17], among other strategies. In contrast to the bulk of knowledge collected from these host-pathogen interactions, no study has yet addressed how *S*. Typhimurium faces recognition of its peptidoglycan (PG) when persisting intracellularly despite the presence of cytosolic immune receptors that recognize molecular patterns of this cell wall component [18]. This lack of information is relevant since *S*. Typhimurium releases massive amounts of outer membrane vesicles (OMVs) while residing within the phagosome [19]. These OMVs shed outside the phagosomal compartment may have as cargo peptidoglycan fragments present in the periplasm of intracellular bacteria. Whether these OMVs stimulate immune defenses from the intracellular environment, is unknown.

The main cell wall component is the PG [20–22], with a basic structure consisting of glycan chains of alternating *N*-acetyl-glucosamine (NAG) and *N*-acetyl-muramic acid (NAM) residues linked by β-(1–4) bonds. The D-lactoyl group of each NAM residue is bound to a stem peptide composed by L-alanine (L-Ala)-D-glutamic acid-(γ)-[D-Glu-(γ)]-a diamino acid-D-alanine (D-Ala)-D-Ala. The diamino acid at the third position is usually lysine in most Gram-positive bacteria and *meso*-diaminopimelic acid (*m*-DAP) in most Gram-negative bacteria. Variations of the basic PG structure have been reported in many bacterial pathogens under laboratory conditions [23,24]. Many of these modifications are linked to immune evasion and, when lacking, pathogenicity is often attenuated [18,25]. PG modifications known to facilitate infection include glycolylation, acetylation or deacetylation of the sugars, and amidation of D-Glu-(γ) or *m*-DAP [26–30]. Some pathogens replace the terminal D-Ala of the stem peptide by non-canonical D-amino acids to adapt their cell wall to stationary phase as an strategy in interspecies competition [31–33] or as mechanism of antibiotic resistance [34].

A distinctive feature of the PG metabolism is the structural remodeling that is constantly exerted by endogenous degradative enzymes. Some of these enzymes (e.g. muramidases, lytic transglycosylases) cleave β-(1–4) glycosidic bonds in the pre-existing PG thus controlling length of the glycan chains, while other enzymes trim the stem peptides regulating crosslinking and allowing the incorporation of the new material required for cell growth. These latter activities are classified as L,D-, D,L- or D,D- endo- or carboxy-peptidases depending on their stereospecificity and the position in the stem peptide of the bond that is cleaved [22,35].

Our previous studies in the PG isolated from *S*. Typhimurium growing actively inside epithelial cells revealed increased cleavage of the NAM-L-Ala amide bond [36]. Cleavage of this bond results in atypical crosslinked muropeptides lacking one of the two NAG-NAM disaccharide units. Such activity in uncrosslinked muropeptides was proposed as potential source of free peptides [36]. Subsequent studies demonstrated that molecular patterns as D-Glu-(γ)-*m*-DAP present in these stem peptides stimulate innate immune defenses. This dipeptide is recognized by NOD1, member of the nucleotide-binding oligomerization domain-like receptor (NLR) family [37,38].

While *S*. Typhimurium proliferates actively within most cultured epithelial cell lines and also *in vivo* inside enterocytes extruded to the intestinal lumen [39], it behaves differently in macrophages and fibroblasts, in which non-proliferating persistent bacterial populations emerge [4,40,41]. Non-proliferating bacteria have also been recently reported to evolve in epithelial cells [42]. In fibroblasts, *S*. Typhimurium undergoes an initial phase of moderate proliferation that is followed by a stage of no increase in the population of intracellular bacteria [7,40,43,44]. Studies in cultured fibroblasts revealed a single round of autophagy attack as a mechanism triggered by the pathogen to reduce its intracellular progeny [43]. This results in a stable and homogeneous pathogen population with an average of 2–3 bacteria per infected cell at 24 h post infection.

Recently, we reported that non-proliferating intracellular *S*. Typhimurium up-regulates inside fibroblasts the D,L-endopeptidase EcgA, which cleaves the D-Glu-(γ)-*m*-DAP motif [45]. Single cell-based analyses also showed that non-proliferating intracellular *S*. Typhimurium attenuates translocation to the nucleus of the pro-inflammatory regulator NF-κB [46]. These findings indicated that the PG of these intracellular bacteria could modulate negatively immune signaling and prompted us to characterize their PG architecture to better understand how *S*. Typhimurium succeeds to persist intracellularly.

Here, we apply ultra-sensitive liquid chromatography coupled to untargeted mass spectrometry to overcome the roadblock of limited material obtained from intracellular bacteria. Our results show an unprecedented complexity in the muropeptide profile and identified an unusual modification in the fourth position of stem peptides present in the PG of non-proliferating intracellular bacteria.

## Results

### Untargeted MS/MS unveils specific muropeptides in the peptidoglycan of non-proliferating intracellular *S*. Typhimurium

As PG metabolism is modulated during growth arrest (e.g. stationary phase), we hypothesized that the reduced growth rate exhibited by *S*. Typhimurium within fibroblasts could encompass structural changes. Given the scarce material that can be obtained from non-proliferating intracellular bacteria [7,47], we drew upon ultra-sensitive UPLC chromatography coupled to untargeted MS/MS. Two fibroblast cell lines, BJ-5ta and NRK-49F, of human and rat origin respectively, were used [4,40]. As additional control, we analyzed the PG of bacteria grown in Luria-Bertani (LB) nutritional medium under the conditions used for infection, overnight culture in static (non-shaking) conditions (S1 Fig).

**Fig 1:**
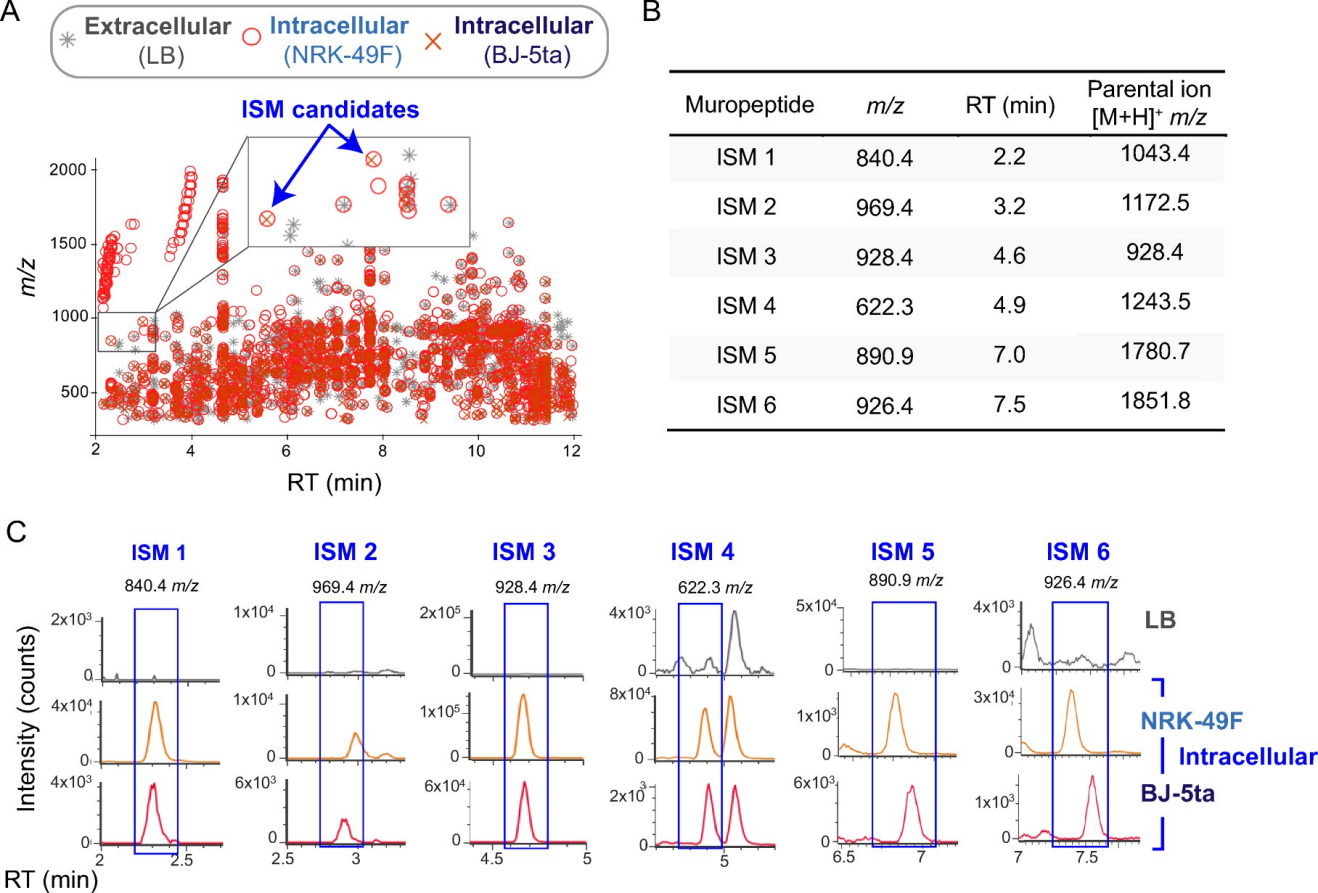
Comparative untargeted MS analysis of PG isolated from *S*. Typhimurium growing in extracellular and intracellular conditions. (A) Chart used for selection of Intracellular-*Salmonella* Muropeptide (ISM) candidates showing the *m/z* values obtained by untargeted MS and their corresponding retention time (RT) of the indicated samples. (B, C) ISM candidates enriched in PG samples of intracellular bacteria that were confirmed by comparative ion chromatographic analyses.

All the *m/z* values and their corresponding retention times obtained for the extracellular and intracellular samples were plotted on the same chart (Fig 1A). The subset of *m/z* present in the two intracellular samples (non-proliferating bacteria collected from NRK-49F and BJ-5ta fibroblasts at 24 h post-infection) but not detected in the PG of bacteria grown in LB, were selected as Intracellular-*S**almonella*-Muropeptides candidates, hereinafter refereed as ISM (Fig 1A). The comparison of the extracted ion chromatogram for all candidates (S1 Table) showed the presence of six ISM (Figs 1B and 1C and S2). ISM 1 to 6 were confirmed as *bona fide* muropeptides as all presented in their fragmentation spectra the loss of 203.07 Da corresponding to the NAG sugar moiety. We further focused on these six ISM that, as shown below, were grouped into two categories according to their structure: (i) products resulting from PG-trimming activities on dimeric muropeptides presenting an L,D-crosslink and, (ii) muropeptides presenting a previously unknown modification in the stem peptide.

### The PG of non-proliferating intracellular *S*. Typhimurium is trimmed at the stem peptides by cleavages affecting molecular patterns important for immune signaling

**Fig 2:**
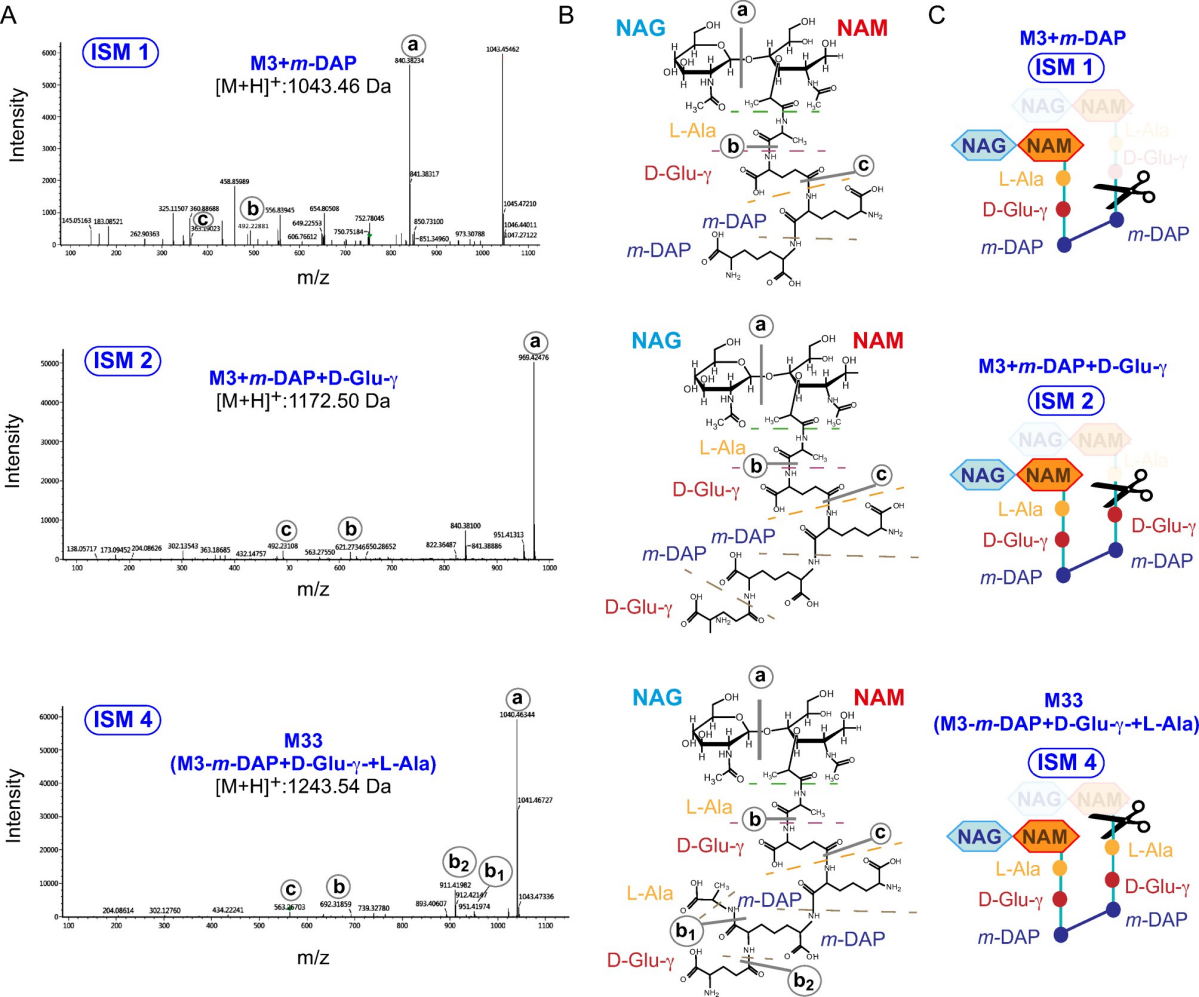
Identification in the PG of intracellular *S*. Typhimurium of muropeptides derived from the action of PG-cleaving enzymes on L,D-crosslinked muropeptides. (A) Fragmentation patterns were obtained by targeted MS of the indicated ions. Theoretical values calculated for the parental ions are shown. (B) Chemical structure determined for each muropeptide and (C) schematic representation of the cleaving activity on a D33 dimer resulting in each of the ISM muropeptides, are shown.

The fragmentation spectra resulting from the targeted MS/MS analysis of the ISM-1, ISM-2 and ISM-4 muropeptides showed structures compatible with the hydrolysis of L,D-crosslinked dimeric species at different positions of the stem peptide (Fig 2A, 2B and 2C). Detection of these muropeptides therefore uncovered increased trimming activity in the PG of non-proliferating *S*. Typhimurium. Our previous studies showed that EcgA, which cleaves the D-Glu-(γ)-*m-*DAP bond, is strongly upregulated by intracellular *S*. Typhimurium persisting inside fibroblasts [45]. The structure of the ISM-1 molecule is the product of such D,L-endopeptidase activity (Fig 2C), suggesting that EcgA could be potentially involved in the generation of this muropeptide. The enzymatic activities needed for the formation of these atypical ISM-1, ISM-2 and ISM-4 muropeptides therefore involve cleavage of molecular patterns like D-Glu-(γ)-*m-*DAP or NAM-L-Ala-D-Glu(γ)-*m-*DAP (Fig 2C). These patterns activate members of the NLR family such as NOD1 and NOD2, this latter requiring in addition the sugar moiety (NAM) bound to the stem peptide [37,48].

### L,D-crosslink (DAP-DAP bridge) is favored in the PG editing occurring in non-proliferating intracellular *S*. Typhimurium

Quantification of the distinct types of muropeptides in extra- and intracellular *S*. Typhimurium (S2 Table) revealed that non-proliferating bacteria residing inside fibroblasts remodel the PG by increasing by ~30% the percentage of L,D-crosslinked muropeptides (Table 1). The relative abundance of muropeptides with this L,D-bridge changed from 5.93% in extracellular bacteria to 8.47% and 7.61% in intracellular bacteria isolated at 24 h post-infection from NRK-49F and BJ-5ta fibroblasts, respectively (Table 1). Importantly, the “atypical” ISM muropeptides (Fig 1B and 1C) increase notoriously in non-proliferating intracellular bacteria, from undetected in extracellular bacteria to ~1% of the total content for the muropeptides with trimmed stem peptides (ISM-1, ISM-2, ISM-4) and, slightly higher than 2% for the ISM muropeptides chemically modified at the terminal position of the stem peptide (ISM-3, ISM-5, ISM-6) (Table 1). Another relevant feature observed in the PG of non-proliferating intracellular bacteria was the shortening in the glycan chain length, from ~53 units in extracellular bacteria to ~37–40 disaccharide units in bacteria isolated from infected fibroblasts (Table 1).

**Table 1:** Quantification of structural parameters in the PG of extracellular and non-proliferating intracellular *S*. Typhimurium.

	Area (%)		
	Extracellular	Intracellular	
	LB	NRK-49F	BJ-5ta
**Monomers**	58.07	60.30	59.21
**Dimers**	39.67	37.36	38.17
**Trimers**	2.27	2.35	2.62
**Lipoprotein**	4.49	3.82	2.80
**Anhydro**	2.40	3.26	3.11
**L,D-crosslinked muropeptides**	5.93	8.47	7.61
**ISM 1,2,4 (D33 trimmed products)**	0.00	0.96	0.77
**ISM 3,5,6 (X-modified muropeptides)**	0.01	2.10	2.32
** **			
**Crosslinkage %**	27.14	25.61	26.48
**Average length glycan chains**	52.92	37.47	40.73

**Fig 3:**
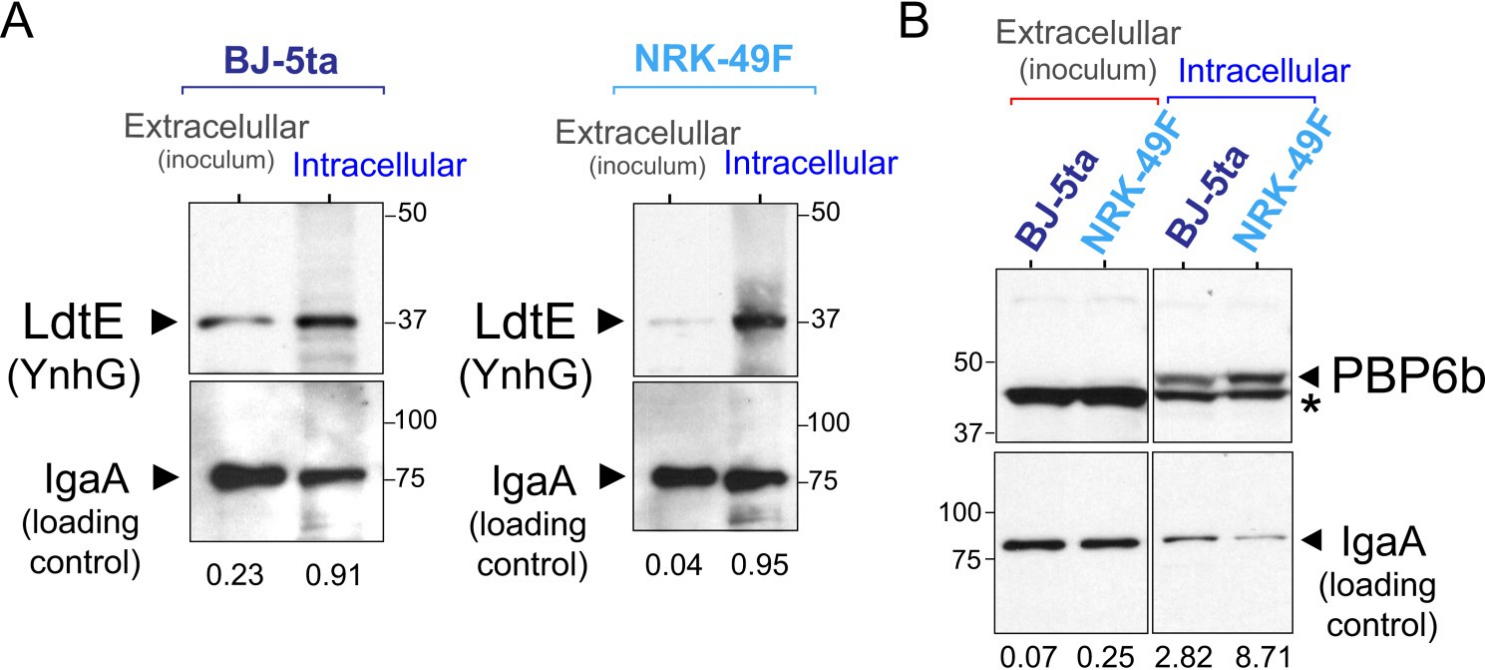
LdtE and PBP6b are up-regulated by non-proliferating intracellular *S*. Typhimurium. Western blots showing the increased amount of (A) the L,D-transpeptidase LdtE (YnhG) and (B) the D,D-carboxypeptidase PBP6b (DacD) in intracellular bacteria. The inner membrane protein IgaA was used in all cases as loading control. In B, the asterisk indicates a different unrelated tagged protein. The data shown correspond to a representative experiment of a total of three independent biological replicates. Numbers indicate for each sample the ratio between the signal measured by densitometry for the band of the protein of interest versus that of the loading control. Size of molecular weight markers (in kDa) are also shown.

The increment in the relative amounts of L,D-crosslinked muropeptides led us to search for the levels of the L,D-transpeptidases LdtD (YcbB) and LdtE (YnhG), known to catalyze this reaction in *Escherichia coli* [49]. We engineered *S*. Typhimurium strains bearing chromosomally-3xFLAG tagged *ynhG* and *ycbB* alleles. These tagged strains were used to infect fibroblasts and to monitor the relative levels of the enzymes in intracellular bacteria at 24 h post-infection. LdtD-3xFLAG was detected at such low levels in extra- or intracellular conditions that prevented analysis and it was not further characterized. Conversely, LdtE-3xFLAG levels notoriously increased in non-proliferating intracellular bacteria compared to extracellular bacteria, up to ~4-fold and ~25-fold in bacteria colonizing BJ5-ta and NRK-49F fibroblasts, respectively (Fig 3A).

We next evaluated whether D,D-carboxypeptidases, cleaving the terminal D-Ala-D-Ala bridge of peptide stems, could also contribute to the increased levels of L,D-crosslinked muropeptides detected in non-proliferating intracellular *S*. Typhimurium. Cleavage of the terminal D-Ala of pentapeptide-muropeptides by D,D-carboxypeptidases decreases the amount of the substrate required for D,D-transpeptidases to form the canonical D,D-crosslink (D-Ala-*m*-DAP bridge) while at the same time increases the tetrapeptide donor substrate for L,D-transpeptidation [50]. The D,D-carboxypeptidase PBP6b, encoded by the *dacD* gene, was recently shown in *E*. *coli* to be a specialized enzyme with higher activity at acidic pH [51]. As the phagosome containing *S*. Typhimurium has an acid pH [52], we measured the levels of PBP6b under non-proliferative intracellular growth conditions. Samples obtained after infecting fibroblasts with a *S*. Typhimurium *dacD*::3xFLAG tagged strain showed that this D,D-carboxypeptidase, almost undetectable in extracellular bacteria, was visualized at high levels in intracellular bacteria irrespective of the fibroblast cell line used. The increase in the relative levels of PBP6b compared to extracellular bacteria was ~35- and ~40-fold for intracellular bacteria collected in NRK-49F or BJ-5ta fibroblasts, respectively (Fig 3B). Taken together, these results indicated upregulation of L,D-transpeptidases and D,D-carboxypeptidases, an alteration that may favor the formation of L,D-crosslinked PG in non-proliferating intracellular *S*. Typhimurium.

### The PG of non-proliferating intracellular *S*. Typhimurium contains unprecedented chemical modifications

**Fig 4:**
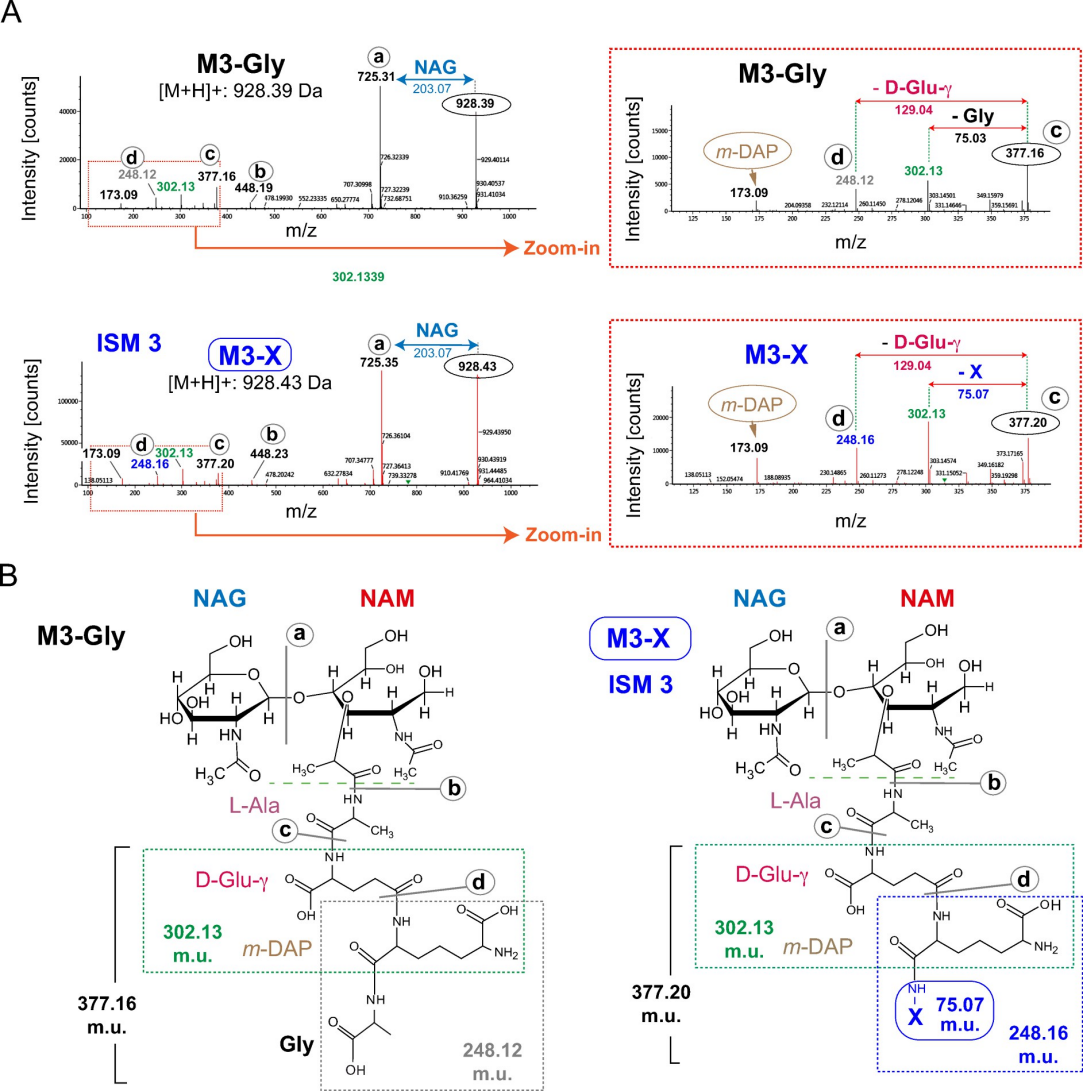
Structure proposed for the muropeptide M3-X identified in the PG of intracellular *S*. Typhimurium. (A) MS/MS fragmentation spectra of ISM 3 (M3-X). For a detailed analysis of the fragmentation pattern, the MS/MS spectra of the molecule of interest were compared with the one obtained from the previously characterized M3-Gly (a muropeptide presenting Gly instead of D-Ala at the fourth terminal position of the stem peptide). Note the ~0.04 Da difference corresponding the C-terminal residue of the stem tetrapeptide. (B) Chemical structure of the two muropeptides.

The second group of muropeptides enriched in the PG of non-proliferating intracellular *S*. Typhimurium accounted for ISM-3, ISM-5 and ISM-6. Their fragmentation spectra indicated that they consist of a family of muropeptides (M3-X, D33-X and D43-X) characterized for having the same unknown molecule (X) replacing D-Ala at the fourth position of the peptide moiety (Figs 4 and S3). A comparison of the fragmentation spectra of ISM-3 with those of previously characterized muropeptides presenting at the terminal position of the stem tetrapeptide a molecule different than D-Ala (in particular glycine, Gly), suggested the presence of a distinct unique moiety as compound X (Fig 4A). Thus, the mass of this compound X of yet unknown nature was 0.04 mass units (m.u.) higher than the molecular weight of Gly (Fig 4A and 4B). Since the MS/MS fragmentation spectra for the M3-Gly muropeptide showed a value of 75.03 Da for the loss of the terminal Gly (75.07 Da) (Fig 4A and 4B), the loss of 75.07 Da in M3-X was therefore compatible with X having a molecular weight of 75.11 Da. The fragmentation spectra of muropeptides D33-X and D43-X (ISM-5 and ISM-6) were also compatible with the presence of a moiety of 75.11 Da as terminal residue of the stem tetrapeptide (S3 Fig). Based on these observations, we searched in the NIST Webbook server (https://webbook.nist.gov/chemistry/mw-ser/) for candidates of a mass of 75.11 Da for the compound X. This search identified alaninol [2-amino-propan-1-ol, (C_3_H_9_NO)] and its stereoisomer 1-amino-2-propan-1-ol (C_3_H_9_NO), with a molecular mass of 75.1097.

Standard protocols for PG analysis by liquid chromatography include a final reduction step using sodium borohydride (NaBH_4_). This step converts NAM residues to NAc-muraminitol, thereby preventing mutarotation of the non-reduced glycan ends that otherwise would result in double peaks in the chromatograms. As chiral amino alcohols can be produced by the reduction of α-amino acids [53–55], we considered the possibility of ISM-3, ISM-5 and ISM-6 resulting from the reduction of the free carboxylic group in the terminal D-Ala of tetrapeptides (S4 Fig). To rule out this possibility, new PG samples were prepared from extracellular and intracellular bacteria, digested with muramidase and divided in two aliquots of which only one was reduced. Compounds matching the expected *m/z* value of M3-X(alaninol) (ISM-3), were detected irrespective of the NaBH_4_ treatment (S4 Fig) supporting the “biological origin” of the X-containing muropeptides. Although some traces of M3-X(alaninol) were detected in the extracellular sample (~0.01%), it was notably enriched in the intracellular sample reaching an estimate of 1.5% of the total PG content (S2 Table). This difference therefore accounted for ~150-fold increase of the ISM-3 [M3-X(alaninol)] muropeptide in non-proliferating intracellular *S*. Typhimurium. Altogether, these data indicated that during the non-proliferative intracellular stage, *S*. Typhimurium favours the synthesis of muropeptides containing alaninol as terminal residue of the stem tetrapeptide.

To further characterize this subset of ISM muropeptides (M3-alaninol, D33-alaninol, D43-alaninol) and once demonstrated their biological origin, we moved to preparative HPLC for their purification. For this, the HPLC chromatogram profile for the PG of non-proliferating intracellular *S*. Typhimurium was compared with two control samples: extracellular bacteria grown in LB medium and in the tissue culture medium (DMEM) in which the fibroblasts were propagated (S5A and S5B and S5C Fig). The collection and further MS/MS analysis of peaks present only in the intracellular sample revealed that the molecules present at the minor peaks 14 and 16 of the HPLC chromatogram corresponded with M3-alaninol and D33-alaninol muropeptides, respectively (S5D and S5E and S5F Fig). These observations confirmed previous MS data and supported the unique chemical modification resulting in alaninol in a fraction of the PG stem peptides of non-proliferating intracellular *S*. Typhimurium. Unfortunately, the extremely scarce amounts of PG that can be purified from non-proliferating intracellular bacteria precluded further studies of these muropeptides by nuclear magnetic resonance or for their use in *in vitro* tests involving stimulation of immune defenses.

### The L,D-transpeptidases LdtD and LdtE generate the atypical crosslinked muropeptides ISM-1, ISM-2 and ISM-4 in intracellular *S*. Typhimurium

The atypical crosslinked muropeptides ISM-1, ISM-2 and ISM-4 identified in intracellular *S*. Typhimurium (Fig 2) have modifications compatible with four enzymatic activities. Firstly, all these muropeptides share a L,D-crosslink (DAP-DAP bridge), a bond that in *E*. *coli* is catalysed by the L,D-transpeptidases LdtD and LdtE [56]. In addition, ISM-1 results from cleavage of the NAM-L-Ala bond known to have as candidate processing enzymes the amidases AmiA, AmiB or AmiC [22,57]. ISM-2 formation results from cleavage of the L-Ala-D-Glu bond, an L,D-endopeptidase activity not yet identified neither in *E*. *coli* nor in *S*. Typhimurium. ISM-4 is generated by a D,L-endopeptidase acting on the D-Glu-(γ)-*m-*DAP bond, with two candidate enzymes exhibiting such activity, NlpC [58] and EcgA [45], this latter absent in *E*. *coli*. Finally, we also considered, based on the up-regulation of PBP6b by non-proliferating intracellular *S*. Typhimurium (see Fig 3), that increased activity of D,D-carboxypeptidases could favor the L,D-crosslink (DAP-DAP bridge).

To establish causality between the modifications detected in the PG of intracellular *S*. Typhimurium and defined enzymes, we generated a collection of isogenic multiple mutants defective in candidate enzymes. The enzyme(s) cleaving the L-Ala-D-Glu(γ) bond to generate the ISM-2 muropeptide, and the enzymatic activities contributing to the modification of the terminal D-alanine to alaninol (ISM-3, ISM-5 and ISM-6 muropeptides), are yet unknown. Regarding the other activities, the multiple deficiency in *S*. Typhimurium of candidates like the amidases AmiA/AmiB/AmiC (cleavage of NAM-L-Ala) or the D,D-carboxypeptidases PBP4/PBP5/PBP6 /PBP6b/PBP7 (cleavage of D-Ala-D-Ala in stem peptide), results in gross morphological changes that prevent their usage in infection assays of cultured mammalian cells. Our observations are consistent with the morphological alterations reported in *E*. *coli* mutants lacking some of the AmiA/AmiB/AmiC amidases [57,59] or multiple D,D-carboxypeptidases [51]. Therefore, before isolating PG material from bacteria recovered from intracellular infections, we focused on those *S*. Typhimurium mutants exhibiting normal rod shape and capacity to invade cultured fibroblasts: i) Δ*ycbB* Δ*ynhG*; ii) Δ*amiA* Δ*amiC*; iii) Δ*amiA* Δ*amiB*; and, iv) Δ*dacC* Δ*dacD*. This latter double mutant was selected among other possible defects in D,D-carboxypeptidases given the high levels of PBP6 and PBP6b detected in non-proliferating intracellular bacteria (Figs 3 and S6).

**Fig 5:**
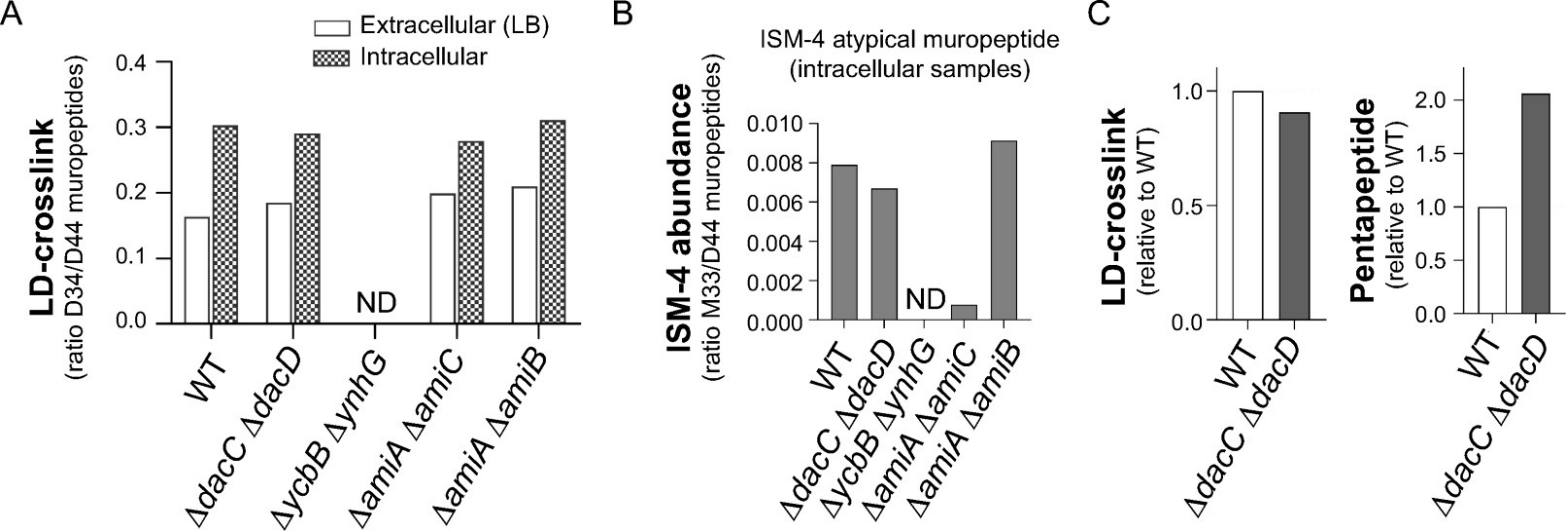
Detection of ISMs in the PG of intracellular *S*. Typhimurium mutant strains lacking enzymes candidate for the formation of atypical muropeptides. (A) Level of L,D-crosslink in the PG of indicated strains growing in LB medium or intracellularly in BJ5-ta human fibroblasts. (B) Detection of the most abundant ISM trimmed muropeptide (ISM-4) in intracellular PG of the indicated strains. For normalization, detected amounts of the M33 muropeptide are shown relative to the amount of D44 present in each sample. (C) Relative amount of L,D-crosslink and pentapeptides detected in the PG of the Δ*dacC*Δ*dacD* mutant recovered from the intracellular infection compared with the PG of intracellular wild type bacteria. ND, not detected. The samples correspond to peptidoglycan purified from intracellular bacteria that were pooled from a minimum of three biological replicates, each one consisting of a minimum of eight 500 cm^2^ flaks.

PG was isolated from these mutants in extracellular conditions (LB medium, stationary phase) and from intracellular bacteria at 24 h post-infection of fibroblasts. The relative level of L,D-crosslink was unaltered in all strains tested except for the Δ*ycbB* Δ*ynhG* double mutant, which shows no presence of L,D-crosslinked muropeptides in extracellular or intracellular conditions (Fig 5A). This result indicated that LdtD and LdtE, but no other L,D-transpeptidase, generate the DAP-DAP bridges in the PG of intracellular bacteria. Consistently, neither ISM-1/ISM-2 nor the most abundant ISM-4 atypical muropeptide were identified in the PG of the *ΔycbB ΔynhG* double mutant recovered from the intracellular infection while they were detected in all the other tested strains. With regards to the PG isolated from the other mutants in intracellular infection conditions, a reduction in ISM-4 was observed in the Δ*amiA* Δ*amiC* mutant, a change not seen in the Δ*amiA* Δ*amiB* mutant (Fig 5B). These findings support AmiC as the preferred amidase that might be used by non-proliferating intracellular *S*. Typhimurium to generate the atypical ISM-4 crosslinked muropeptide. On the other hand, a minor decrease of L,D-crosslinking was observed in the PG of intracellular Δ*dacC* Δ*dacD* mutant bacteria (Fig 5C), consistent with a slight increase in pentapeptide muropeptides in the PG of this mutant following the infection of fibroblasts (Fig 5C). This finding supports a possible role of PBP6 and/or PBP6b in the increase of L,D-crosslink observed in the PG of non-proliferating bacteria. Collectively, the structural analysis of the PG isolated from mutants defective in candidate enzymes after infecting cultured fibroblasts proved that no L,D-transpeptidase apart from LdtD and LdtE generates the DAP-DAP bridge present in intracellular bacteria (and therefore in the atypical ISM-1, ISM-2 and ISM-4 L,D-crosslinked muropeptides), whereas the amidase AmiC could be responsible for cleaving the NAM-L-Ala bond that generates ISM-4.

### The modifications attaining for the generation of the ISM-1, ISM-2 and ISM-4 muropeptides are dispensable for attenuating immune response in the fibroblast

**Fig 6:**
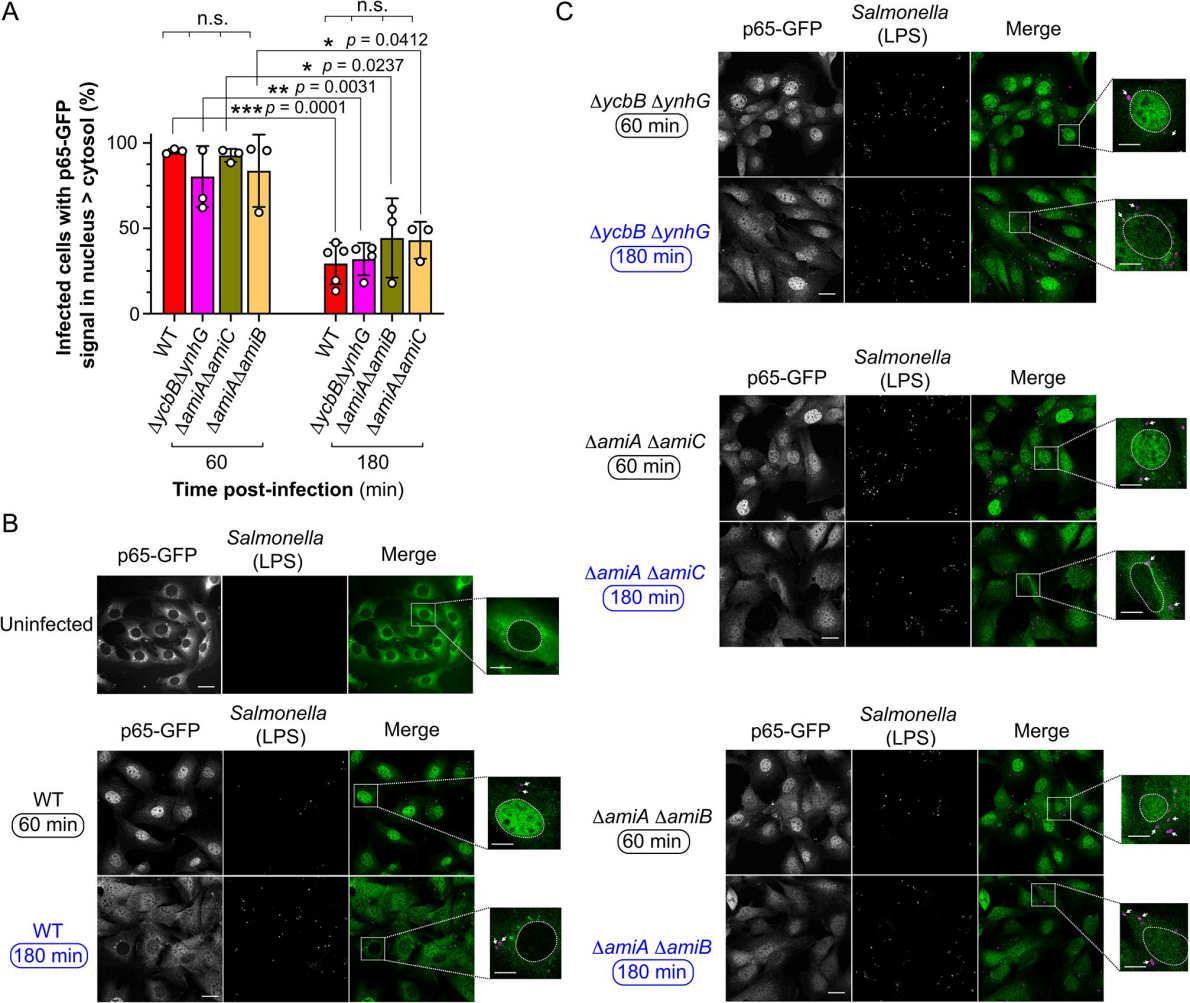
The deficiency in enzymes candidates to generate ISM-1, ISM-2 and ISM-4 does not impair intracellular *S*. Typhimurium for attenuating the NF-κB response. (A) Percentage of infected fibroblasts having higher fluorescence signal in the nucleus derived from the p65-GFP reporter. Data are shown for two infection times (60, 180 min) and wild type and the indicated double mutants. *, *P* = 0.01 to 0.05; **, *P* = 0.001 to 0.01; ***, *P* = 0.0001 to 0.001. Unpaired Student *t* test for comparison of same strain in two different post-infection times and one way ANOVA with multiple comparisons of strains at the same post-infection time. Data from independent replicates are shown by circles and bars represent the mean and standard deviation values. (B) Mouse reporter fibroblasts expressing p65-GFP uninfected or infected with wild type *S*. Typhimurium at two post-infection times, 60 and 180 min. Representative images of p65-GFP signal (green) and *Salmonella* stained with anti-LPS antibodies (magenta). Images on the right are enlargements of individual uninfected or infected cells. Arrows point to intracellular bacteria. Bars, 25 μm (left) and 10 μm (enlargements). (C) Same as in wild type bacteria but for the indicated double mutants.

Our previous studies showed that upon entry into cultured fibroblasts, *S*. Typhimurium stimulates the pro-inflammatory regulator NF-κB in an initial phase that is followed by attenuation of the immune response in the infected cell as intracellular bacteria adapt to the non-proliferative state [46]. We reasoned that the modifications found in the PG of non-proliferating intracellular *S*. Typhimurium could be potentially linked to this attenuation of the immune response. To test this, mouse embryonic fibroblasts from knock-in mice expressing p65-GFP, a fluorescent reporter with the NF-κB p65 subunit fused to GFP, were challenged with the different mutants defective in L,D-crosslinking (Δ*ycbB* Δ*ynhG*) or amidases cleaving the NAM-L-Ala bond (Δ*amiA* Δ*amiC* and Δ*amiC* Δ*amiB*). Cytosol to nucleus translocation of NF-κB p65 was monitored in the population of infected cells at two post-infection times, 60 and 180 min. At early times, all the mutants stimulate NF-κB nuclear translocation and further attenuate the response at later times with rates comparable to those of wild type bacteria (Fig 6A). A similar behavior of these mutants was confirmed by microscopy analyses examining in parallel a culture of naïve uninfected fibroblasts as negative control (Fig 6B and 6C). Collectively, these results suggested that increase of L,D-crosslink and the modifications involving the generation of the atypical crosslinked muropeptides ISM-1, ISM-2 and ISM-4 bearing L,D- (DAP-DAP) bridges (Fig 2) could be dispensable for attenuating the immune response in the fibroblast infection model.

### Alaninol-modified tetrapeptides exhibit limited immuno-stimulatory capacity

The structural analyses of PG isolated from all mutants lacking candidate enzymes in intracellular infections showed the presence of alaninol-modified ISM atypical muropeptides like ISM-3 (S7 Fig). This result supported the existence of yet unknown enzyme(s) responsible for the D-alanine to alaninol modification. The fact that these mutants attenuate NF-κB nuclear translocation at late infection times (Fig 6) and that all of them modify D-Ala to alaninol in fourth position of stem peptides (S7 Fig), led us to propose this unprecedented modification as responsible for diminishing the immuno-stimulatory level of the PG of intracellular bacteria. Although D-Ala in fourth position has been suggested to diminish NOD1 recognition by comparing the stimulatory effect of [NAM-L-Ala-D-Glu(γ)-*m-*DAP] versus that of [NAM-L-Ala-D-Glu(γ)-*m*-DAP-D-Ala] [37], no study has yet compared the effect of sugar-free peptides with the terminal D-Ala as only variation. This action could be relevant if free peptides bearing this modification were released from macromolecular PG by an NAM-L-Ala amidase acting on atypical “uncrosslinked” muropeptides.

**Fig 7:**
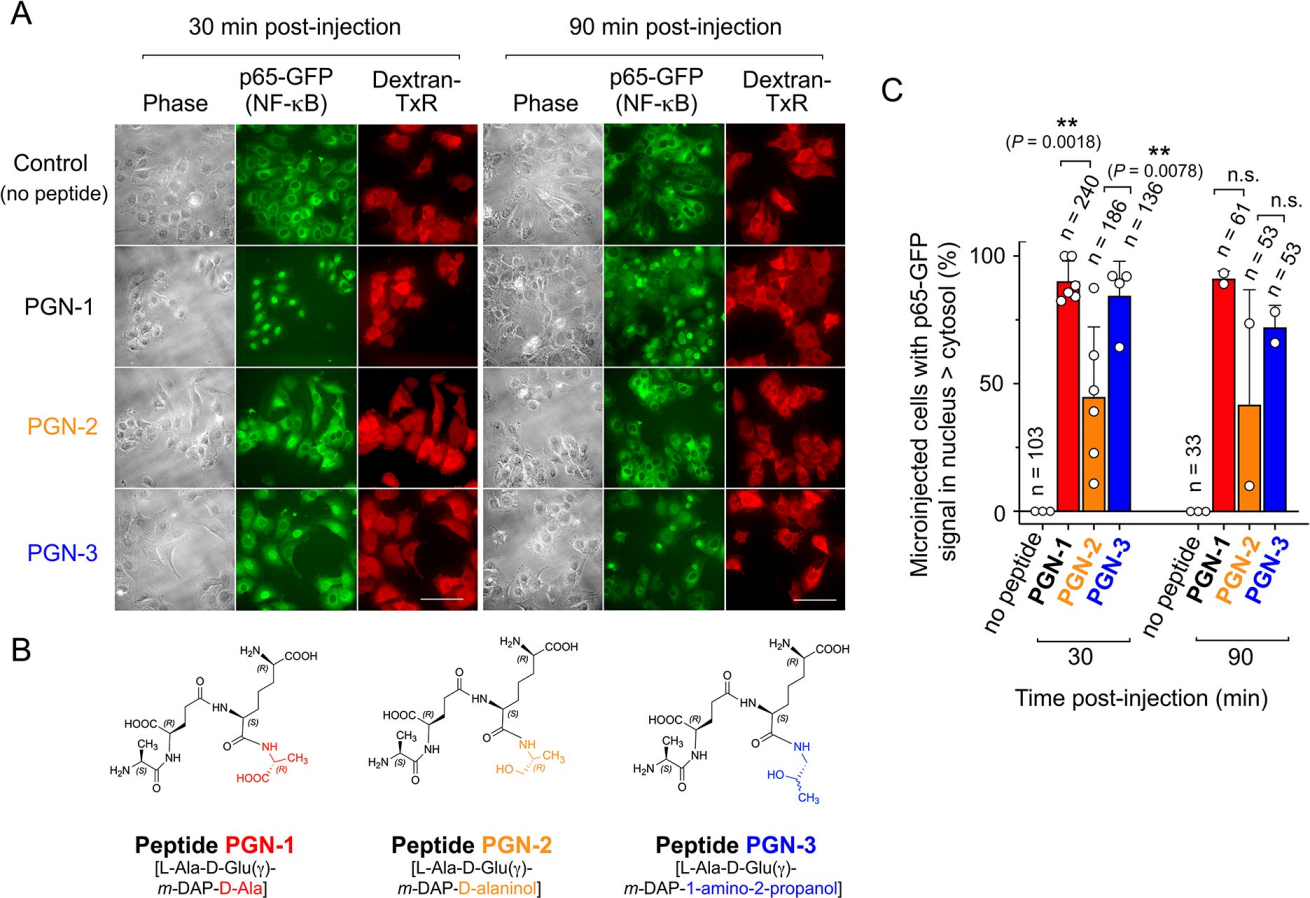
The presence of alaninol at the C-terminal residue of a canonical tetrapeptide (substituting D-Ala) attenuates stimulation of the pro-inflammatory regulator NF-κB. (A) Images of mouse embryonic fibroblasts from knock-in mice expressing the reporter p65-GFP, with p65 being a subunit of NF-κB. Dextran Texas Red was added to the peptide solution to identify at single-cell level the microinjected cells. Two post-injection times, 30 and 90 min, are shown. The images are of a representative assay of a minimum of three independent biological replicates. Bar, 10 μm. (B) Structure of the three synthetic tetrapeptides used, PGN-1, PGN-2 and PGN-3. (C) Quantification of the percentage of microinjected cells having a higher p65-GFP signal in nucleus versus the cytosol. The circles are quantifications corresponding to independent biological replicates with *n* referring to the total number of microinjected cells examined. Bars show median and standard error values. **, *P* = 0.001 to 0.01; n.s., not significant, by one way ANOVA with multiple comparisons.

As the enzyme(s) responsible for the D-Ala to alaninol modification are still unknown with no chance to reproduce such modification *in vitro*, and the impossibility of obtaining enough highly pure PG material from non-proliferating intracellular *S*. Typhimurium, we chemically synthesized three distinct tetrapeptides (PGN-1, PGN-2 and PGN-3) with the sequence L-Ala-D-Glu-(γ)-*m-*DAP-**Y**, in which **Y** was = D-Ala (natural sequence); = D-alaninol (2-amino-propan-1-ol), or = 1-amino-2-propanol, respectively. These tetrapeptides were micro-injected into the mouse embryonic reporter fibroblasts expressing p65-GFP. The presence of D-alaninol as fourth residue (peptide PGN-2) decreased dramatically its capacity to trigger translocation of p65-GFP from the cytosol to the nucleus, a proxy for NF-κB stimulation (Fig 7A). Conversely, the presence of 1-amino-propanol in the C-terminus of the tetrapeptide (PGN-3) reduced minimally the capacity to stimulate NF-κB, whereas peptide PGN-1, bearing D-Ala as fourth residue, activated NF-κB in all the micro-injected cells (Fig 7A and 7B and 7C). Quantification of percentage of microinjected cells showing differential fluorescent signal in nucleus versus cytosol proved these differences as statistically significant (Fig 7C). Furthermore, the fact that PGN-1 with sequence L-Ala-D-Glu(γ)-*m-*DAP-D-Ala stimulated translocation of NF-κB to the nucleus denoted a distinct biological effect compared to NAM-[L-Ala-D-Glu(γ)-*m-*DAP-D-Ala], previously reported not to be stimulatory [37]. Taken together, these data proved that the substitution of D-Ala by an amino alcohol in the C-terminus of the PG stem peptide affects immune recognition of sugar-free peptides bearing the stimulatory pattern D-Glu(γ)-*m-*DAP.

## Discussion

To our knowledge, this study represents the first comprehensive structural analysis of PG purified from intracellular bacteria during a persistent infection using highly sensitive chromatographic techniques. The absence of comparable studies might be explained by the difficulty in isolating sufficient amounts of PG material from such a reduced number of intracellular bacteria [60]. Despite descriptions of muropeptide structures existing for some obligate intracellular pathogens like *Chlamydia trachomatis*, *Coxiella burnetii* or *Mycobacterium leprae*, these studies reported a relatively low number of muropeptide species [61–63]. Interestingly, these analyses provided evidence of atypical modifications like the substitution of L-Ala by Gly in intracellular chlamydiae and *M*. *leprae* or the amidation of D-Glu in *M*. *leprae* [64]. Whether these pathogens exploit such modifications to evade immune signalling was tested only in the case of *M*. *leprae*, in which no effect of these atypical muropeptides on NOD2 recognition, was observed [28].

The highly sensitive untargeted MS technology uncovered two unexpected groups of muropeptide structures enriched in the PG of non-proliferating intracellular *S*. Typhimurium isolated from fibroblasts. Remarkably, these muropeptides were not identified in this same pathogen proliferating actively within epithelial cells, in which the PG is subjected to substantial trimming by a NAM-L-Ala amidase that recognizes exclusively D,D-crosslinked muropeptides [36]. This is a marked difference with the non-proliferative stage, in which an NAM-L-Ala amidase that recognizes L,D-crosslinked muropeptides is favored, yielding the ISM-4 muropeptide identified in our study (Fig 2C). The data reported here with *S*. Typhimurium mutants lacking defined amidases point to AmiC as the preferred enzyme exploited by non-proliferating intracellular *S*. Typhimurium for this reaction. Future studies involving *in vitro* assays with purified amidases and distinct muropeptide species as substrates could shed light into this apparent specificity of the amidase for D,D- or L,D-crosslinked substrates.

Another feature unique for the state of no proliferation was the presence of products of endopeptidases cleaving the D-Glu(γ)-*m-*DAP and L-Ala-D-Glu(γ) bonds in stem peptides of L,D-crosslinked muropeptides that results in the atypical ISM-1 and ISM-2 muropeptides, respectively (Fig 2C). These endopeptidase activities could be of outmost importance for the pathogen. Thus, they could warrant that pro-inflammatory stem peptides bearing the D-Glu(γ)-*m-*DAP motif [37] are not released from macromolecular PG if an NAM-L-Ala amidase acts on uncrosslinked muropeptides. This is certainly an area for future research, to dissect the nature of the muropeptides that the pathogen sheds while residing in the intracellular niche, technically challenging but needed to decipher how intracellular bacteria interfere with immune signalling.

The L,D-crosslinkage is known to be favored in the PG of mycobacteria, prone to cause intracellular infections [65], and the obligate intracellular pathogen *Coxiella* spp. [61]. At present, we do not have an obvious reason to explain why the L,D-crosslink is present in all the unusual trimmed-muropeptides detected in non-proliferating intracellular *S*. Typhimurim (ISM-1, ISM-2 and ISM-4) (Fig 2C). It is plaussible that the PG-remodeling enzymes acting in intracellular non-proliferating *S*. Typhimurim prefer the less abundant L,D-crosslinked muropeptides as substrates.

The absence of L,D-crosslinked muropeptides in the PG of the *S*. Typhimurium Δ*ycbB*Δ*ynhG* mutant, in both nutrient media and in the intracellular niche, demonstrates that no L,D-transpeptidase additional to LdtD and LdtE is involved in forming L,D-crosslink in intracellular bacteria and, therefore, the atypical ISM-1, ISM-2 and ISM-4 crosslinked muropeptides. LdtE is up-regulated by non-proliferating intracellular *S*. Typhimurium whereas LdtD is basically undetected, so we assign to LdtE a more important role as enzyme responsible for the L,D-transpeptidation activity in the PG of non-proliferating intracellular bacteria. L,D-transpeptidation plays also a central role in the pathogenesis of the human-adapted serovar *S*. Typhi. Intriguingly, *ynhG* is a pseudogene in *S*. Typhi and LdtD (YcbB) is the enzyme that edits PG in this serovar in such a manner that allows secretion of the typhoid toxin by intracellular bacteria [66]. The apparent opposite usage of L,D-transpeptidases that form DAP-DAP bridges by *S*. Typhimurium and *S*. Typhi, which display disparate intracellular phenotypes and have distinct host tropism [67,68], supports PG editing as a key modulator of the intracellular lifestyle of *S*. *enterica* serovars.

The marked increase of the D,D-carboxypeptidase PBP6b in intracellular *S*. Typhimurium, an enzyme with higher activity in acidic enviroments [51], could also be determinant to increase the formation of the L,D-crosslinked muropeptides inside the host cell. Interestingly, PG built with higher content of L,D-bridge contributes to withstand alterations in the outer membrane [69]. This opens the possibity of enhaced L,D-transpeptidation as mechanism to counterbalance the potential damage of shedding of OMVs and concomitant loss of membrane material by intra-phagosomal *S*. Typhimurium [19].

An important result derived from the double mutants used in the infection assays was that none of them failed in modifying the C-terminal residue of stem tetrapeptides for an amino alcohol compatible with alaninol. The scarcity of the PG material that can be obtained from non-proliferating intracellular *S*. Typhimurm precluded the definition by nuclear magnetic resonance of the exact stereochemistry of this amino alcohol of 75.1097 mass units. However, the chirality of the central carbon atom of the alaninol, absent in the central C atom of 1-amino-2-propan-1-ol, allow us to assign alaninol as most probable molecule occupying this position in the atypical ISM-3 (M3-X) muropeptide. Thus, chirality is important for the formation of the peptide bond. The presence of alaninol in the PG of non-proliferating intracellular *S*. Typhimurium was infered to have consequences in terms of immune evasion based on two evidences. First, all the mutants tested attenaute NF-κB nuclear translocation at late infection times with rates comparable to wild type bacteria (Fig 6). Second, a synthetic tetrapeptide with D-alaninol in the four position, and not its stereoisomer 1-amino-2-propanol, reduced significantly NF-κB stimulation following its microinjection into reporter fibroblasts (Fig 7). We therefore suggest that D-alaninol as fourth residue in the stem peptide could impact immune recognition despite the presence of the immuno-stimulatory D-Glu(γ)-*m-*DAP motif in second and third positions. Future studies directed to examine the stimulatory potential of stem peptides of distinc lenghts and bearing D-Ala or D-alaninol as terminal carboxy residue, could provide further support to our model. Note that in the synthesis of peptides we prefered the esteroisomer D-alaninol versus L-alaninol since the natural residue in that position is D-Ala. We have also preliminary data showing that the addition of D-alaninol, but not that of L-alaninol, limits the capacity of *S*. Typhimurium to grow in nutrient rich media, inferring some damage to the cell envelope when added in excess, probably by disregulation of enzymes acting on the PG. The apparent more inocuous L-alaninol is indeed present in a glycopeptidolipid unrelated to PG that is abundant in the envelope of the opportunistic pathogen *Mycobacterium avium* [70]. So, besides its potential role as immuno-modulatory molecule, D-alaninol could also limit growth inside the host cell, a central objetive for the pathogen that establishes a persistent infection.

**Fig 8:**
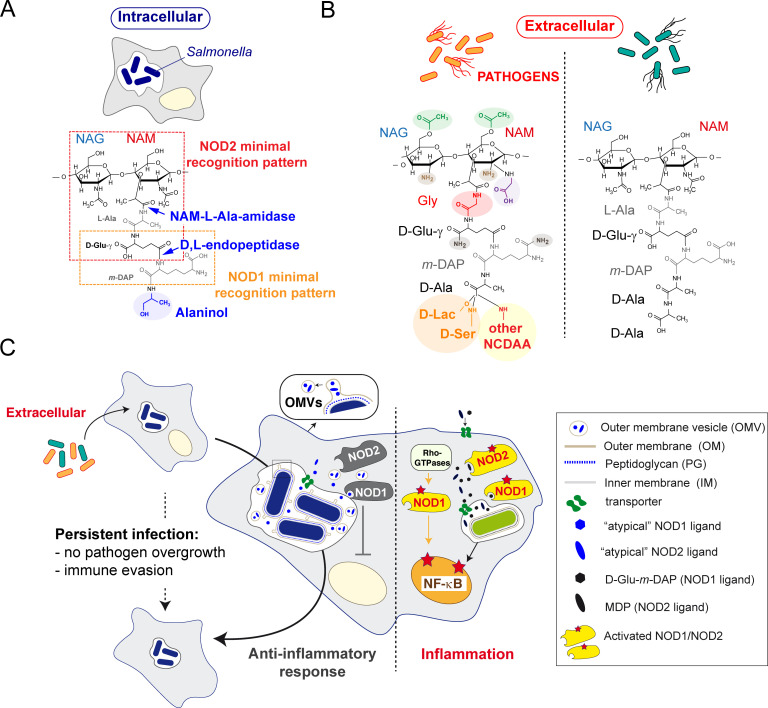
Working model depicting how PG editing in non-proliferating intracellular *S*. Typhimurium interferes with immune signaling and promotes persistence. (A-B) Comparison of structural alterations identified in the PG of intracellular *S*. Typhimurium versus those reported in other pathogens under laboratory conditions and the structure of a canonical muropeptide of Gram-negative bacteria (extracellular); (C) Scheme that shows how the release from intra-phagosomal *S*. Typhimurium of outer membrane vesicles having as cargo atypical PG fragments, like those containing D-alaninol, could favor immune evasion and persistence by interfering with NOD1/NOD2 responses. The model compares such event with an infection in which *S*. Typhimurium releases pro-inflammatory (canonical) PG fragments that stimulate NF-κB. The model also integrates the NOD1 activation by pathogen effectors that target Rho-GTPases independently of PG fragments reported by others [77]. How the PG fragments are transported from the phagosome or vesicles to the cytosol remains poorly understood. NAG: *N*-acetylglucosamine, NAM: *N*-acetylmuramic acid, *m-*DAP: *meso*-diaminopimelic acid; D-Ser: D-serine, D-Lac: D-lactate, NCDAA: non-canonical D-amino acid; OMV: outer membrane vesicles.

Collectively, our data establish three levels of modifications in the PG of non-proliferating intracellular *S*. Typhimrium. Two of them attain for the increase of L,D-crosslink together with the group of atypical L,D-crosslinked ISM-1, ISM-2 and ISM-4 muropeptides, modifications that may contribute to withstanding envelope damage in the harsh intra-phagosomal niche and with and additional potential to limit immune recognition. The third group would be the atypical muropeptides containing alaninol, which could contribute directly to attenuate immune recognition. The PG editing reported here in non-proliferating intracellular *S*. Typhimurium differs from structural alterations known to promote evasion of innate defenses or antibiotic resistance in other pathogens and that involve mainly changes in the sugar moeities or substitutions of terminal D-Ala by non-canonical D-amino acids or D-lactate (Fig 8). A major challenge for future studies is to identify the enzyme(s) responsible for the PG editing involving the presence of D-alaninol, which may be considered a new signature for the intracellular lifestyle of non-proliferating *S*. Typhimurium.

## Materials and methods

### Bacteria and eukaryotic cell growth and infection conditions

The *S*. Typhimurium strains and plasmids used in this study are listed in S3 Table. Bacteria were grown overnight at 37°C without shaking in LB nutrient rich medium. Under this growth conditions, *S*. Typhimurium is highly competent for invasion of non-phagocytic cells [71]. BJ-5ta human fibroblasts (ATCC CRL 4001) and NRK-49F rat fibroblasts (ATCC CRL-1570) were propagated in Dulbecco’s Modified Eagle’s Medium (DMEM), 10% (v/v) fetal bovine serum at 37°C in a 5%CO_2_ atmosphere, as described [72]. BJ-5ta human fibroblasts were supplemented with 20% (v/v) Medium 199. Large scale infection conditions of fibroblasts seeded in Nunc Square 500-cm^2^ Bio Assay dishes (ref. 166508, ThermoFisher Sci.) were performed as described elsewhere [7,36]. For specific experiments, requiring large quantities of peptidoglycan from intracellular bacteria to obtain enough material of purified muropeptides for subsequent spectrometry analyses, fibroblasts were infected with the *phoP7953*::Tn*10* mutant, which proliferates at high rate inside this host cell type [44]. Control assays showed that those muropeptides subjected to mass spectrometry analyses were shared by wild type and *phoP7953*::Tn*10* isogenic strains.

### DNA techniques

Genetic manipulation for gene inactivation was carried out as described previously [73]. 3xFLAG epitope tagging was performed using the method of Uzzau et al. [74]. Oligonucleotides and plasmids used in this study are listed in S4 Table.

### Purification of PG

Murein sacculi isolation from extracellular and intracellular bacteria was performed as previously described [36], with a few modifications. Briefly, at the desired post-infection times (normally 24 h) the infected fibroblasts were scraped in a 15 mL solution containing 1% (v/v) phenol, 19% (v/v) ethanol, 0.4% (w/v) SDS and 1 μg/mL DNAse. These conditions led to selective lysis of the fibroblasts while preserving bacterial integrity, as previously reported [7]. This 15 mL sample was centrifugated (18,000 x *g*, 30 min, 4°C) to obtain a sediment. The bacterial pellets from a minimum of 24 Bio-Assay 500-cm^2^ dishes were pooled by resuspension in a final total volume of 3 mL of phosphate buffered saline (PBS), pH 7.4. For those experiments requiring larger amounts of PG, the number of Bio-Assay 500-cm^2^ dishes containing fibroblasts were increased up to 140, with a proportional final volume for the pooled pellets. This sample containing the pooled bacteria was gently added drop by drop to an equal volume of boiling 8% SDS and the solution -maintained in closed glass tubes to avoid evaporation- shaken during a minimum of 8 h at 90°C. The sample was then shaken for additional 18 h at room temperature. The sacculi were recovered by high-speed centrifugation (346,200 x *g*, 20 min, 23°C in an Optima MAX-UP ultracentrifuge (Beckman Coulter) and washed with MilliQ H_2_O. Clean sacculi were digested with 1 μg trypsin for 5 h at 37°C. Trypsin digestion results in cleavage of Braun’s lipoprotein between Lys(K)46 and Tyr(Y)47 residues, leaving a Tyr-Arg-Lys (Y-R-K) tripeptide bound to the *m-*DAP residue of muropeptides covalently bound to this lipoprotein. The trypsin-treated sacculi were finally recovered by high-speed centrifugation (346,200 x *g*, 20 min, 23°C). Clean sacculi were digested with muramidase (100 μg/ml) for 16 h at 37°C. After boiling for protein inactivation, insoluble material was pelleted by centrifugation and discarded. Muropeptides in the soluble fraction were reduced with 0.5 M sodium borate pH 9.5 and sodium borohydride (10 mg/mL final concentration, 30 min at room temperature). The pH of the samples was adjusted to 3.5 with orthophosphoric acid before liquid chromatography analysis.

### High-resolution muropeptide analysis

Muropeptide composition was assessed on an Ultra-Performance Liquid Chromatography system interfaced with a Xevo G2/XS Q-TOF mass spectrometer (Waters Corp.). Chromatographic separation was achieved using an ACQUITY UPLC BEH C18 Column (2.1 mm × 150 mm, 1.7 μm pore size. Waters Corp.) heated at 45°C. 0.1% formic acid in Milli-Q water (buffer A) 0.1% formic acid in acetonitrile (buffer B) were used as eluents. The gradient of buffer B was set as follows: 0–3 min 5%, 3–6 min 5–6.8%, 6–7.5 min 6.8–9%, 7.5–9 min 9–14%, 9–11 min 14–20%, 11–12 min hold at 20% with a flow rate of 0.175 ml/min; 12–12.10 min 20–90%, 12.1–13.5 min hold at 90%, 13.5–13.6 min 90–2%, 13.6–16 min hold at 2% with a flow rate of 0.3 mL/min; and then 16–18 min hold at 2% with a flow rate of 0.25 mL/min. Chromatograms were recorded at 204 nm. The QTOF-MS instrument was operated in positive ionization mode.

When untargeted MS/MS was performed, the parameters set for ESI were: capillary voltage at 3.0 kV, source temperature to 120°C, desolvation temperature to 350°C, sample cone voltage to 40 V, cone gas flow 100 L/h and desolvation gas flow 500 L/h. For targeted MS, the collision energy was set to scan between 6 eV and 15–40 eV. Mass spectra were acquired at a speed of 0.25 s/scan and the scans were in a range of 100–2000 *m/z*. Data acquisition and processing were performed using UNIFI software package (Waters Corp.). When needed the molecular structure of each muropeptide was obtained by using ChemSketch (ACD/ChemSketch, Advanced Chemistry Development, Inc. Toronto, Canada, 2013).

### Characterization of Intracellular *Salmonella* muropeptides (ISM)

Mass to charge ratio (*m/z*) values presenting between 400–2,000 m.u. and with retention times within 2 and 12 minutes were extracted from the untargeted MS/MS data of the samples to create x-y plots showing *m/z* values (x-axis) against their respective retention time (RT, y-axis) by using MATLAB. Then the 3 graphs were overlapped creating one that was visually examined for the selection of *m/z* occurring exclusively in intracellular samples at each RT.

As collected *m/z* values from untargeted MS include both precursor and fragment ions data, we first grouped them regarding their observed RT (conceivably ions appearing at the same RT are part of the spectrum of the same molecule). Then, by the analysis of their MS-spectrum we tried to determine if the different *m/z* values corresponded to fragment or adduct ions of the same molecular ion, or to the same ion with a different charge state, thus reducing the list of *m/z* values considered as intracellular-unique-ions to those presented in Fig 1B. The ion chromatograms of these selected values were extracted using the untargeted MS data of the three samples: extracellular and intracellular, NRK-49F and BJ-5ta fibroblasts (Fig 1). The precursor ion of those candidates confirmed to be exclusively intracellular were analyzed by targeted MS/MS (as indicated in high-resolution muropeptide analysis) to determine the chemical structure of the molecules.

### Chromatography analyses by HPLC

Samples of digested PG (muropeptides) were analysed on a nano liquid chromatography system (Eksigent Technologies nanoLC Ultra 1D plus, AB SCIEX, Foster City, CA) coupled to a 5600 Triple TOF mass spectrometer (AB SCIEX, Foster City, CA) with a nano-electrospray ion source. Samples were injected on a C18 PepMap trap column (5 μm, 100 μm I.D. x 2 cm, Thermo Scientific) at 2 μL/min, in 0.1% formic acid in water, and the trap column was switched on-line to a C18 nanoAcquity BEH analytical column (1.7 μm, 100 Å, 75 μm I.D. x15 cm, Waters). Equilibration was done in mobile phase A (0.1% formic acid in water), and peptide elution was achieved in a 40 min linear gradient from 5% - 40% B (0.1% formic acid in acetonitrile) at 250 nL/min. Instrument calibration was continuously updated through automatic injection and calibration of a commercial peptide mixture (Beta-gal tryptic digest, AB SCIEX). Mass accuracy ranged between 5–10 ppm. The mass spectrometer operated in data-dependent acquisition mode. For TOF scans, the accumulation time was set to 250 ms, and per cycle, up to 15 precursor ions were monitored. MS1 and MS2 spectra were analysed manually using PeakView software (SCIEX).

### Isolation of intracellular *S*. Typhimurium for determination of LdtE (YnhG) and PBP6b (DacD) levels

BJ-5ta human fibroblasts and NRK-49F rat fibroblasts were infected with the chromosomally 3xFLAG-tagged isogenic strains MD5225 (*ynhG*::3xFLAG) and MD3790 (*dacD*::3xFLAG *mltF*::3xFLAG *mepA*::3xFLAG *mltB*::3xFLAG-Km) and, as described for peptidoglycan purification, processed following described protocols involving selective lysis of the fibroblasts that preserves bacterial integrity [7]. Samples of intracellular bacteria were obtained at 24 h post-infection and the experiment performed in three biological independent replicates.

### Western-blot immunoassays

Primary antibodies and their working dilutions used for western blotting included: mouse monoclonal anti-FLAG epitope (clone M2, Sigma) 1:5,000 and rabbit polyclonal anti-IgaA 1:10,000 [75]. Goat polyclonal anti-mouse or anti-rabbit IgG conjugated to horseradish peroxidase (Bio-Rad) were used as secondary antibodies at a 1:10,000 dilution. SDS-PAGE and western blotting were performed as described [76].

### Tetrapeptide synthesis

Details regarding the synthesis and characterization of the tetrapeptides PGN-1, PGN-2 and PGN-3 are described in S1 Dataset.

### Monitoring of NF-κB activity in reporter p65-GFP fibroblasts

Mouse embryonic fibroblasts (MEF) expressing p65-GFP were propagated in DMEM medium with 10% FBS, 50 μM -mercaptoethanol, 1% L-glutamine, 1% sodium pyruvate and 1% non-essential amino acids at 37°C in a 5% CO_2_ atmosphere. For the microinjection assays, cells were seeded in μ-Dish 35 mm, high Grid-500 dishes (Ibidi) to locate microinjected cells. A minimum of 100 fibroblasts were microinjected per dish with a mix containing 2 mM of the tetrapepide and 1 mg/mL of 70 kDa lysine fixable Dextran-Texas Red (ThermoFisher Sci.). This counter-stain reagent allowed to identify the microinjected fibroblasts in the grid. A volume of ~ 1–2 nL was microinjected per fibroblast. After microinjection, fibroblasts were transferred to the CO_2_ incubator and maintained at 37°C for 30 or 90 min before fixation with 3% (w/v) paraformaldehyde for 10 min at room temperature. After fixation, cells were washed with PBS pH 7.4 and visualized in a Leica DMi8 S epifluorescence microscope equiped with a Hamamatsu Flash 4 sCMOs digital camera. This experiment was repeated for minimum of three times as independent biological replicates.

### Statistical analysis

Data were analysed using GraphPad Prism, version 8.0, software (GraphPad Inc. San Diego, CA). *t*-test and one-way ANOVA with multiple comparisons were used for data analysis. Significance was established at *P* values ≤ 0.05.

## Supporting information

S1 Fig Muropeptide composition of peptidoglycan (PG) from *S*. Typhimurium growing in LB medium. (A) Total ion chromatogram (TIC) obtained by untargeted MS/MS; (B) muropeptide identification of peak numbers indicated in panel A; (C) schematic representation of each muropeptide structure. NAG: *N*-acetylglucosamine, NAM: *N*-acetylmuramic acid; *m-*DAP: *meso*-diaminopimelic acid; Lpp: Braun’s lipoprotein-oligopeptide remaining bound to the muropeptide after cleavage with trypsin; NAM-Anh: anhydrous form of NAM.
(TIF)

S2 Fig Extracted ion chromatograms of ISM-candidates *m/z* values obtained in the untargeted MS/MS analysis. PG samples obtained from LB cultures (grey chromatogram), and from intracellular bacteria in NRK-49F and BJ-5ta fibroblasts (orange and red chromatograms, respectively). Green vertical rectangles highlight those *m/z* specific to intracellular samples. These ISM candidates are highlighted in red with their respective numbers (ISM 1 to ISM 6). (See also Fig 1C).
(TIF)

S3 Fig **MS/MS fragmentation spectra of ISM 5 (D33-X, panel A) and 6 (D43-X, panel B).** For detailed analysis of the fragmentation patterns, the MS/MS spectra of the molecules of interest were compared with the MS/MS spectra of muropeptides where X corresponds with a Gly. Note that the ~0.02 Da difference observed between the Gly and X-containing muropeptides is coming from the comparison of double charged molecular ions.
(TIF)

S4 Fig The M3-X muropeptide is detected by targeted MS/MS in non-reduced PG samples of non-proliferating intracellular *S*. Typhimurium. (A) Schematic representation of the reduction process of a M4 muropeptide. Sodium borohydride (NaBH_4_) treatment of muramidase-digested PG is intended for the reduction of the NAM sugar (violet arrow and circle) of muropeptides. In the presence of a strong reduction agent, alanine hydrogenation could potentially lead to the production of an amino-alcohol (arrow and circle in red dashed line); (B) Structure of the non-reduced form of the M3-X and theoretical value expected for its parental ion; (C-D) Detection and quantification of M3-X muropeptide by targeted MS/MS present in reduced and non-reduced PG samples of S. Typhimurium. In the extracellular sample, additionally to the M3-X muropeptide (blue peaks), the M3-Gly muropeptide (red peaks) was also detected.
(TIF)

S5 Fig HPLC muropeptide-elution pattern of peptidoglycan (PG) purified from *S*. Typhimurium grown extra- or intracellularly. The conditions tested were: (A) DMEM medium; (B) LB; and (C) non-proliferating intracellular bacteria collected after 24 h of infection of BJ-5ta human fibroblasts. Peaks 14 and 16 correspond to muropeptides detected exclusively in the PG of intracellular bacteria and collected for further analysis. (D) Identity of the muropeptides observed in the HPLC- chromatograms. (E) Peaks 14 and 16 collected and identified by mass spectrometry. Shown are their MS/MS fragmentation spectra. Note that the indicated observed ions for the muropeptides correspond to sodium adducts that match the expected masses plus 22 Da of Na^+^. The peptidoglycan purified from intracellular bacteria that were pooled from a total of sixteen biological replicates, each one consisting of 6–8 culture dishes of 500-cm^2^ (see Materials and methods).
(TIF)

S6 Fig PBP6 (DacC) is detected at high levels in intracellular *S*. Typhimurium. Western blots showing high amounts of the D,D-carboxypeptidase PBP6 (DacC) in intracellular bacteria collected at 24 h post-infection of NRK-49F and BJ-5ta fibroblasts. For comparison, levels of the enzyme in the infecting bacteria grown statically overnight in LB medium (inoculum), are shown. The inner membrane protein IgaA was used in all cases as loading control. The data shown correspond to a representative experiment of a total of three independent biological replicates.
(TIF)

S7 Fig Detection of the most abundant alaninol(X)-modified ISM muropeptide (ISM-3) in the PG of mutant strains recovered from the intracellular infection. For normalization, the amounts of the M3-X are shown relative to those of the M4 muropeptide present in each sample. The PG was purified from intracellular bacteria that were pooled from a minimum of three biological replicates, each one consisting of a minimum of eight dishes of 500-cm^2^ with cultured fibroblasts.
(TIF)

S1 Dataset Chemistry of synthetic tetrapeptides. This dataset includes the synthesis routes, general conditions, experimental protocols, chemical characterization and ^1^HNMR, ^13^CNMR and mass spectra of the tetrapeptides PGN-1, PGN-2 and PGN-3.
(DOCX)

S1 Table *m/z* values obtained in the untargeted MS/MS comparative analysis that include candidates to correspond to Intracellular Salmonella Muropeptides (ISM). (DOCX)

S2 Table Quantification of the most abundant muropeptides detected in the PG samples by untargeted MS/MS. (DOCX)

S3 Table *S*. Typhimurium strains and plasmids used in this study. (DOCX)

S4 Table Oligonucleotides used in this study. (DOCX)
