# Supplementary figures and images for: Peptidoglycan editing in non-proliferating intracellular Salmonella as source of interference with immune signaling
Source: PLoS Pathog. 2022 Jan 25;18(1):e1010241. doi: 10.1371/journal.ppat.1010241 (PMC8815878; doi:10.1371/journal.ppat.1010241)

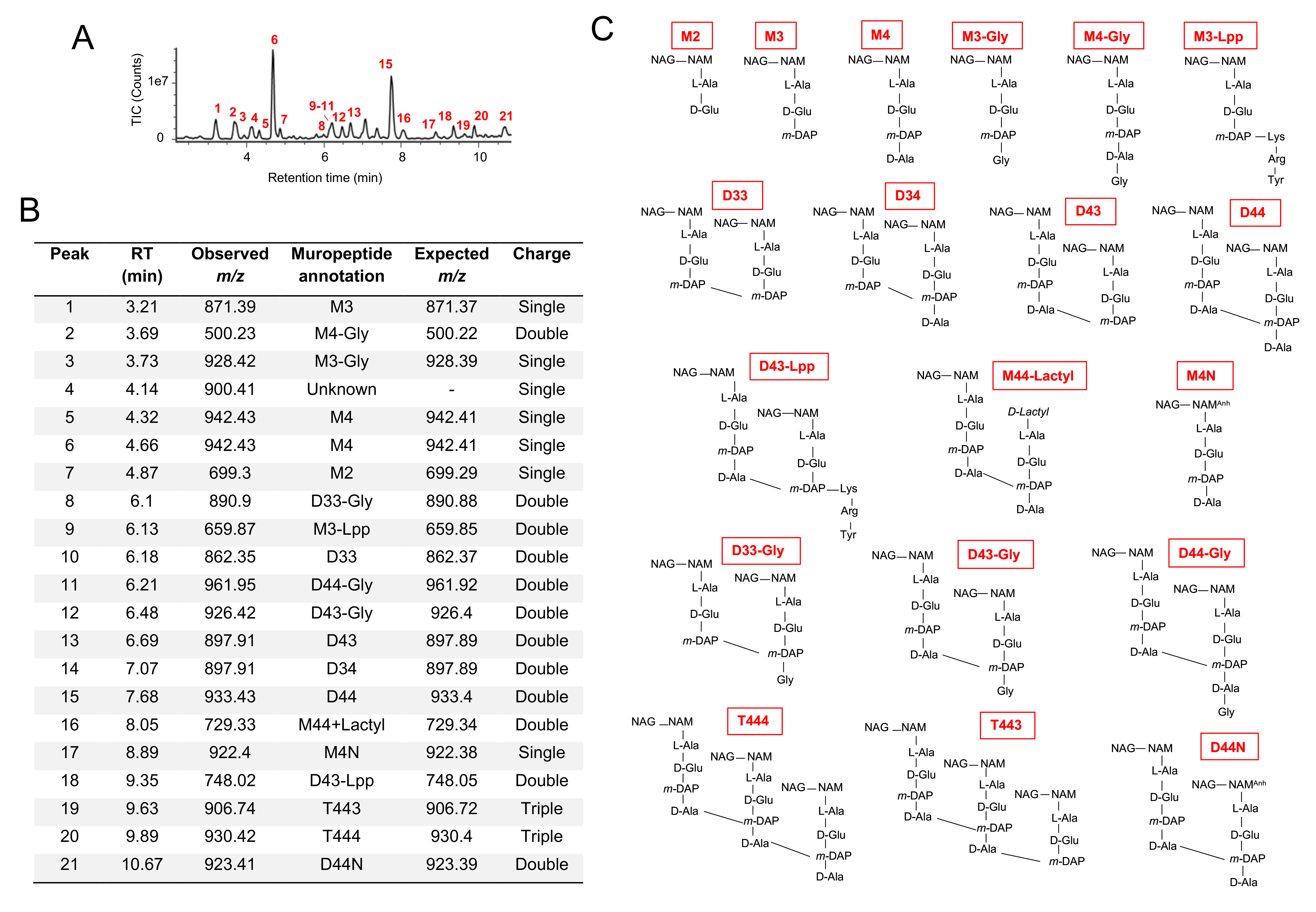

Supplement: S1 Fig — (A) Total ion chromatogram (TIC) obtained by untargeted MS/MS; (B) muropeptide identification of peak numbers indicated in panel A; (C) schematic representation of each muropeptide structure. NAG: N-acetylglucosamine, NAM: N-acetylmuramic acid; m-DAP: meso-diaminopimelic acid; Lpp: Braun’s lipoprotein-oligopeptide remaining bound to the muropeptide after cleavage with trypsin; NAM-Anh: anhydrous form of NAM. (TIF) [file ppat.1010241.s001.tif]

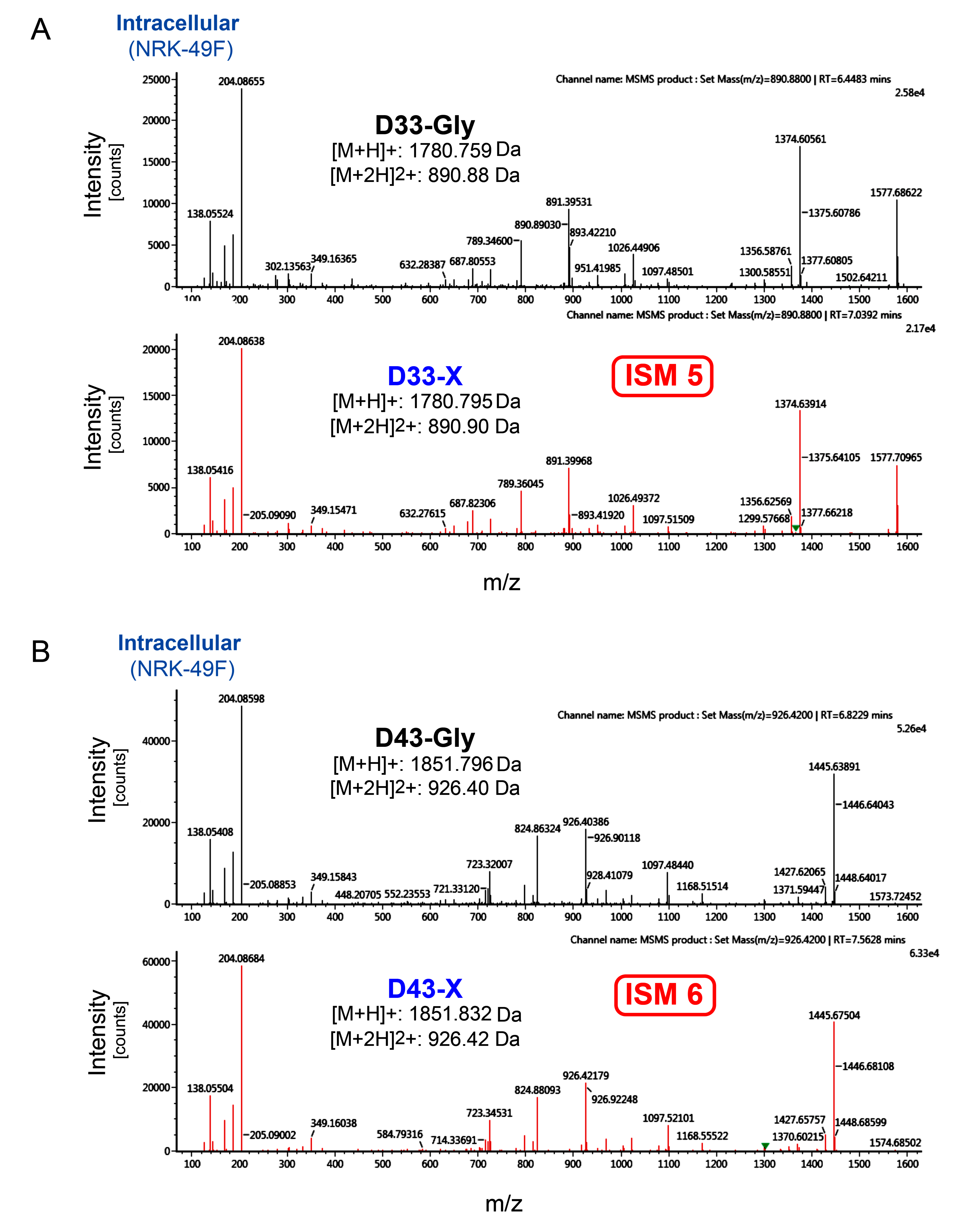

Supplement: S3 Fig — MS/MS fragmentation spectra of ISM 5 (D33-X, panel A) and 6 (D43-X, panel B). For detailed analysis of the fragmentation patterns, the MS/MS spectra of the molecules of interest were compared with the MS/MS spectra of muropeptides where X corresponds with a Gly. Note that the ~0.02 Da difference observed between the Gly and X-containing muropeptides is coming from the comparison of double charged molecular ions. (TIF) [file ppat.1010241.s003.tif]

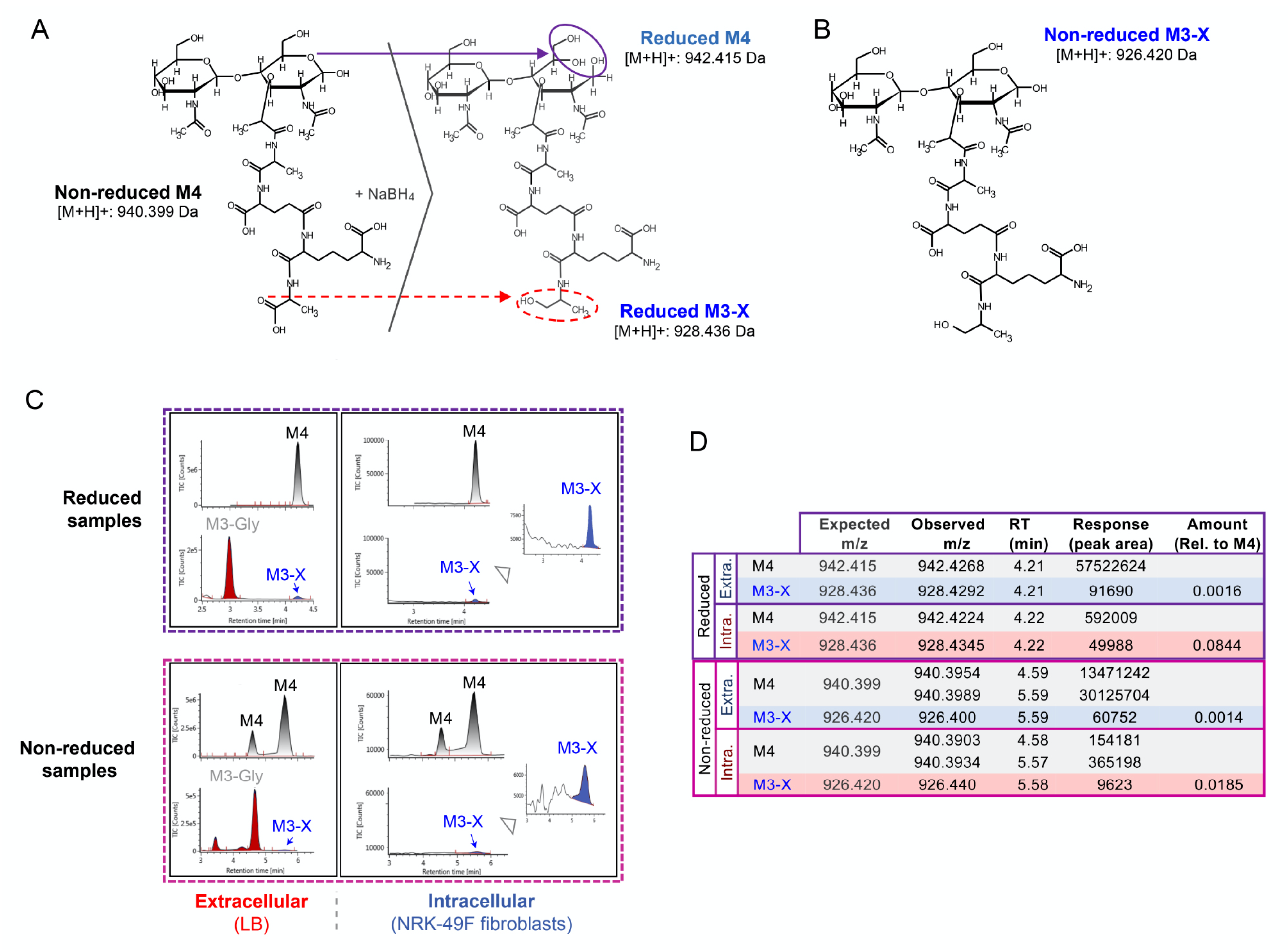

Supplement: S4 Fig — (A) Schematic representation of the reduction process of a M4 muropeptide. Sodium borohydride (NaBH4) treatment of muramidase-digested PG is intended for the reduction of the NAM sugar (violet arrow and circle) of muropeptides. In the presence of a strong reduction agent, alanine hydrogenation could potentially lead to the production of an amino-alcohol (arrow and circle in red dashed line); (B) Structure of the non-reduced form of the M3-X and theoretical value expected for its parental ion; (C-D) Detection and quantification of M3-X muropeptide by targeted MS/MS present in reduced and non-reduced PG samples of S. Typhimurium. In the extracellular sample, additionally to the M3-X muropeptide (blue peaks), the M3-Gly muropeptide (red peaks) was also detected. (TIF) [file ppat.1010241.s004.tif]

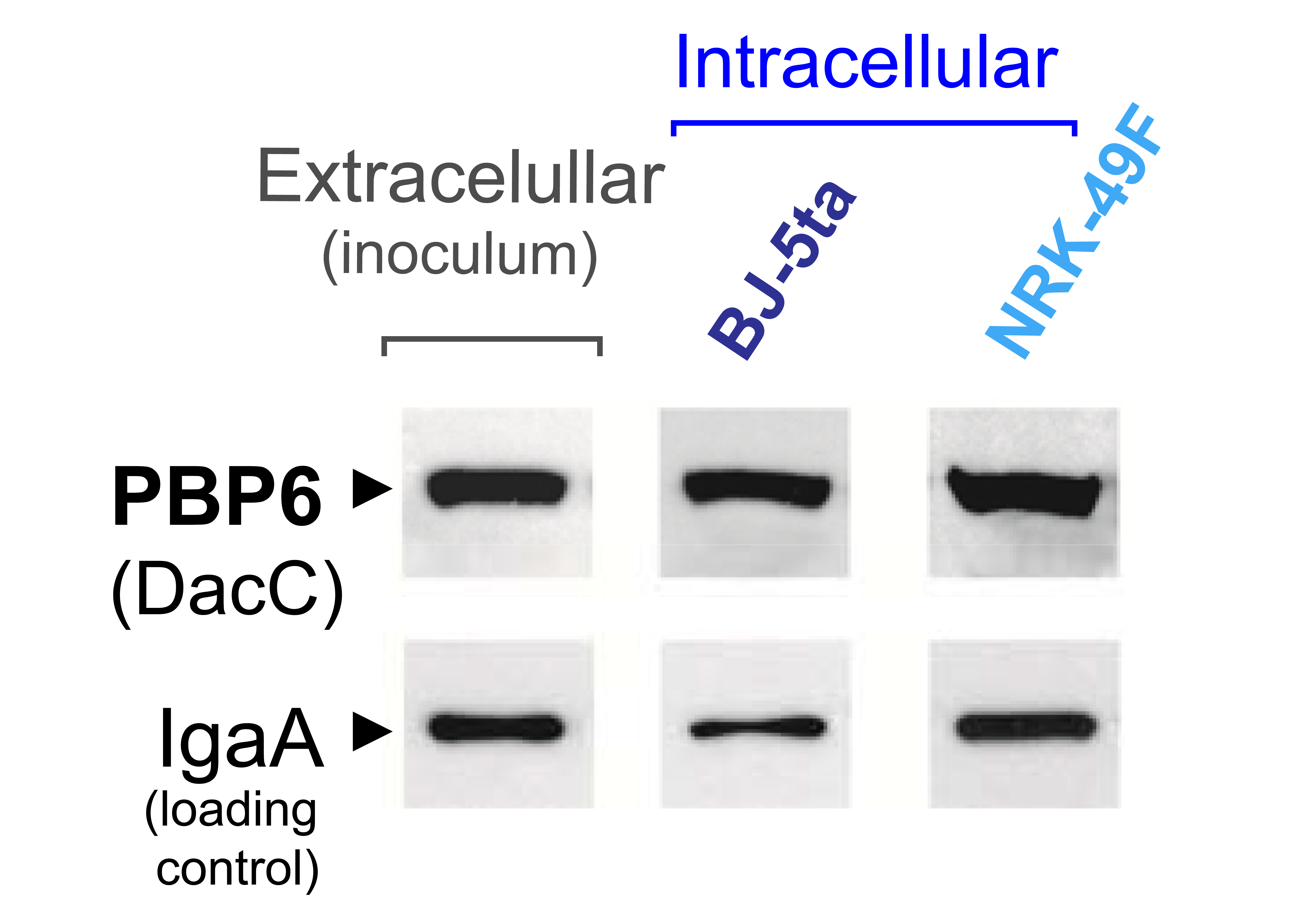

Supplement: S6 Fig — Western blots showing high amounts of the D,D-carboxypeptidase PBP6 (DacC) in intracellular bacteria collected at 24 h post-infection of NRK-49F and BJ-5ta fibroblasts. For comparison, levels of the enzyme in the infecting bacteria grown statically overnight in LB medium (inoculum), are shown. The inner membrane protein IgaA was used in all cases as loading control. The data shown correspond to a representative experiment of a total of three independent biological replicates. (TIF) [file ppat.1010241.s006.tif]

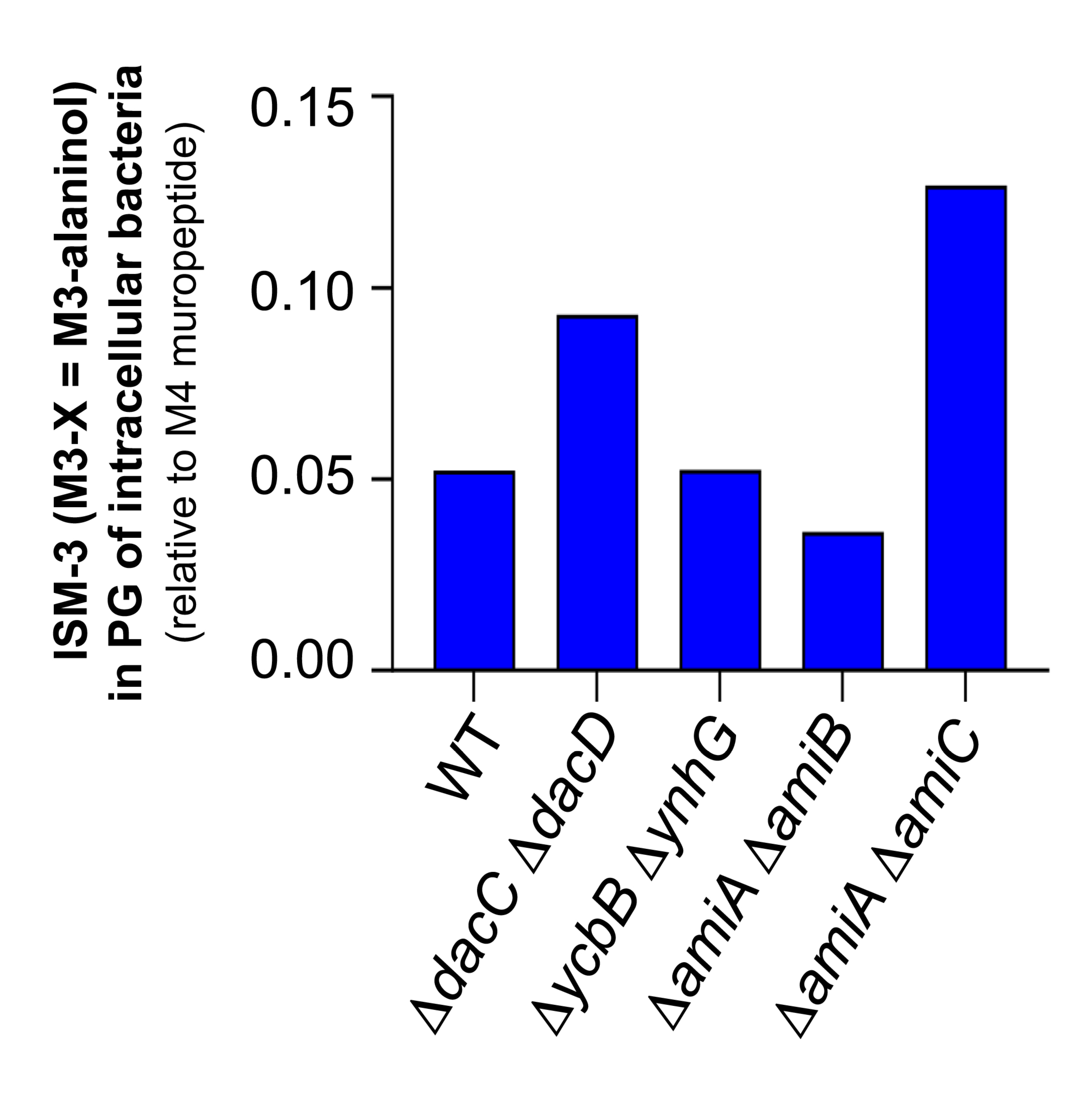

Supplement: S7 Fig — For normalization, the amounts of the M3-X are shown relative to those of the M4 muropeptide present in each sample. The PG was purified from intracellular bacteria that were pooled from a minimum of three biological replicates, each one consisting of a minimum of eight dishes of 500-cm2 with cultured fibroblasts. (TIF) [file ppat.1010241.s007.tif]
