# Supplementary material for: Peptidoglycan editing in non-proliferating intracellular Salmonella as source of interference with immune signaling
Source: PLoS Pathog. 2022 Jan 25;18(1):e1010241. doi: 10.1371/journal.ppat.1010241 (PMC8815878; doi:10.1371/journal.ppat.1010241)
Supplement: S1 Dataset — This dataset includes the synthesis routes, general conditions, experimental protocols, chemical characterization and 1HNMR, 13CNMR and mass spectra of the tetrapeptides PGN-1, PGN-2 and PGN-3. (DOCX) [file ppat.1010241.s008.docx]

**S1 Dataset. Chemistry of synthetic tetrapeptides**

- Synthetic routes
- General conditions
- Experimental protocols and compound characterization
- ^1^HNMR, ^13^CNMR and mass spectra of tetrapeptides PGN-1, PGN-2 and PGN-3
- **Synthetic routes**

Three distinct tetrapeptides PGN-1, PGN-2 and PGN-3 with the sequence L‑Ala-d-Glu (γ)-*m*-DAP-R in which R was = d-Ala (PGN-1), R = d-alaninol (2-amino-propan-1-ol) (PGN-2) or R = 1-amino-2-propanol (PGN-3) (Fig 7B), were synthesized in order to be tested as agonists/antagonists of the Nucleotide-binding oligomerization domain protein 1 (Nod 1), involved in the inflammatory responses against pathogenic microbes. The synthesis of these tetrapeptides is hampered by the difficulty involved in obtaining the *meso*-2,6-diaminopimelic acid (*m*-DAP) fragment, contained in their structures. DAP is an unusual amino acid with two chiral centers with different stereochemistry and two amine and carboxyl functionalities. The synthesis of DAP derivatives represents a great synthetic challenge that implies a careful selection of orthogonally protecting groups of the two carboxylic and amine moieties present in its structure to selectively introduce the desired modifications. In addition, it is crucial the use of adequate reaction conditions to avoid racemization and control the chirality of the target compounds.

Several different synthetic routes to the *m*-DAP moiety have been described [1-9]. In our case, this fragment was prepared according to the method first reported by Boons et al. [1,2] that implies an olefin cross-metathesis reaction [10] between properly protected allyl and vinyl glycine derivatives using Grubb’s second-generation catalyst, followed by reduction of the double bond of the resulting cross coupling product.

The starting allyl- and vinylglycines using in this reaction should be suitably protected in order to ensure that the resulting DAP derivative had an appropriate protecting group pattern for the subsequent synthetic steps.

The synthesis started with (R)-allylglycine whose amino group was protected with Boc-anhydride to give N-Boc (R)-allylglycine 1 (89%) [3] (Scheme 1). Next, esterification of the carboxylic acid, using tert-butyl 2,2,2-tricholoracetimidate [11,12] afforded N-Boc protected tert-butyl ester 2 [13] with 71% yield. Subsequent reaction of 2 with a small excess (2 eq) of the commercially available Cbz-vinylglycine-OMe 3 in the presence of Grubbs’ second-generation catalyst afforded the cross-coupling product 4 in moderate yield 50% (Scheme 1). Catalytic hydrogenation of 4 over Pd/C in ethyl acetate resulted in reduction of the double bond with concomitant removal of the N-benzyloxycarbonyl (Cbz) group to give the DAP derivative 5 (quant %). This key intermediate has an unmasked amino group ideal for further extension.





**Scheme 1.** Synthesis of intermediate 5

Coupling of 5 with Fmoc-D-Glu-OtBu in the presence of 1-[Bis(dimethylamino)methylene]-1H-1,2,3-triazolo[4,5-b]pyridinium-3-oxid hexafluorophosphate (HATU) as coupling reagent and N,N-diisopropylethylamine (DIPEA) as base, gave dipeptide 6 (67%) (Scheme 2). Treatment of 6 with piperidine in DMF (20%), to remove the Fmoc protecting group, afforded an amine intermediate that was immediately reacted, without purification, with Boc-L-Ala-OH in the presence of HATU and DIPEA to give tripeptide 7 (67%). Treatment of 7 with LiOH/H_2_O, followed by acidification at pH = 2, afforded compound 8 in 68% yield, with an unmasked carboxylic acid group, that is the key intermediate in the subsequent steps of the synthesis.





**Scheme 2.** Synthesis of intermediate 8

Then, intermediate 8 was coupled respectively with H-D-Ala-OtBu, tert-butyl D-alaninol and tert-butyl 1-amino-2-propanol in the presence of HATU/DIPEA to afford the corresponding tetrapeptides 9 (68%), 10 (60%) and 11 (65%) (Scheme 3). The subsequent treatment with TFA in dichloromethane (20%), to remove simultaneously the Boc and tert-butyl protecting groups, afforded the target deprotected tetrapeptides PGN-1, PGN-2 and PGN-3 in quantitative yield (Scheme 3). The chloride salts were obtained when a solution of the corresponding peptide in water was treated with several drops of hydrochloric acid (37%) and then lyophilized.





**Scheme 3.** Synthesis of tetrapeptides PGN-1, PGN-2 and PGN-3

The characterization of these compounds was performed by ^1^H and ^13^C NMR spectroscopies and HRMS.

- **General conditions**

Commercial reagents and solvents were used as received from the suppliers without further purification unless otherwise stated. The solvents used in some reactions were dried prior to use. DMF dry was commercially available (Aldrich).

Analytical thin-layer chromatography (TLC) was performed on aluminum plates pre-coated with silica gel 60 (F_254_, 0.20 mm). Products were visualized using an ultraviolet lamp (254 nm and 365 nm) or by heating after treatment with a 5% solution of phosphomolybdic acid (PMA) or vanillin in ethanol.

The compounds were purified by a) flash column chromatography on silica gel (60 Merck 230-400 mesh), b) preparative centrifugal circular thin layer chromatography (CCTLC) on a Chromatotron® (Kiesegel 60 PF_254_ gipshaltig, Merck) with layer thickness of 1 mm and a flow rate of 2–4 mL/min.

For HPLC analysis, an Agilent Technologies 1120 Compact LC with a reverse-phase column ACE 5 C18-300 (4.6 mm x 150 mm, 3.5 μm) equipped with a PDA (Photo Diode Array) detector was used. Acetonitrile was used as mobile phase A, and water 0.05% of TFA was used as mobile phase B with a flow rate of 1 mL·min^-1^. All retention times are quoted in minutes and the gradients are specified for each compound in the experimental data.

For high-resolution mass spectrometry (HRMS) an Agilent 6520 Accurate Mass QTOF (quadrupole time of flight) platform coupled with LC/MS and equipped with an electrospray interface (ESI) working in the positive-ion (ESI+) and negative-ion (ESI-) mode was used.

NMR spectra (^1^H, ^13^C NMR) were recorded on a Varian UNIT INOVA-300 (300 MHz), Bruker AVANCE 300 (300 and 75 MHz), Varian INOVA-400 (400 and 100 MHz), Varian MERCURY-400 (400 and 100 MHz) and Varian-500 (500 and 125 MHz) spectrometers, using (CD_3_)_2_SO and CDCl_3_ as solvents. Chemical shift (δ) values are reported in parts per million (ppm) relative to tetramethylsilane (TMS) in ^1^H and CDCl_3_ (δ = 77.0) in ^13^C NMR. Coupling constant (J values) are reported in hertz (Hz) and multiplicities of signals are indicated by the following symbol: s (singlet), d (doublet), t (triplet), q (quadruplet), m (multiplet) and bs (broad singlet). Some two-dimensional spectra (COSY, HSQC and HMBC) were performed to identify the structure. The final compounds were lyophilized using a Telstar 6-80 system.

- **Experimental protocols and compound characterization**

Boc-(R)-Allylglycine-OH (1) [3]. (R)-2-Allylglycine (1 g, 8.69 mmol, 1 eq) was dissolved in a mixture of 1M NaOH (20 mL) and dioxane (10 mL). Boc anhydride (3.79 g, 17.38 mmol, 2 eq) was added under ice-cooling. The reaction mixture was allowed to warm up to ambient temperature and stirring was continued for 18h. After completion of the reaction, the mixture was concentrated under reduced pressure, and the aqueous phase was washed with diethyl ether to remove the excess of Boc anhydride. The pH was adjusted to 2.5 with HCl, and the mixture was extracted with EtOAc (3 x 20 mL). The combined organic layers were dried over MgSO_4_ and concentrated under reduced pressure. The desired compound (1) was obtained as colorless oil (1.68 g, 89%). Characterization of this compound was consistent with those found in the literature [3].

Boc-(R)-Allylglycine-OtBu (2) [13]. Boc-(R)-AllylGly-OH 1 (935.4 mg, 4.35 mmol, 1 eq) was dissolved in 8 mL of a mixture of anhydrous dichloromethane: tetrahydrofuran (4:1). To the stirring solution, tert-butyl 2,2,2-tricholoracetimidate (3.08 mL, 17.38 mmol, 4 eq) was added. The reaction was stirred at room temperature overnight under argon atmosphere, and then concentrated under reduced pressure. The residue was washed with NaHCO_3_ (3 x 100mL) and brine (3 x 100mL). The organic layer was dried (MgSO_4_), filtrated and concentrated. The residue was precipitated with hexane. The crude product was purified by flash silica gel column chromatography (Hex:AcOEt, 1:1) to afford 845 mg (71%) of 2 as a colorless oil. HPLC [gradient: A:B, 50-100% of A in 10 min]: 5.11 min. Characterization of this compound was consistent with those found in the literature [13].

1-Methyl 7-tert-butyl (2S,6R)-2-(benzyloxycarbonylamino)-6-[(tert-butoxycarbonyl)amino]-hept-3-enedioate (4). A solution of the allylglycine derivative 2 (366 mg, 1.35 mmol) and Cbz-(S)-vinylglycine-OMe 3 (674 mg, 2.70 mmol) in dichloromethane (10 mL) was bubbled with Ar for 5 min, and then Grubbs second generation catalyst (GII) was added (115 mg, 10 mol%). The reaction was stirred at 30 ºC overnight and then concentrated in vacuo. The residue was purified by flash silica gel column chromatography (Hex:EtOAc, 5:1) to afford 336 mg (50%) of 4 as a brown-yellow oil. HPLC [gradient: A:B, 10-100% of A in 10 min]: 9.7 min. ^1^H-RMN (400 MHz, Chloroform-d) δ 7,26 (m, 5H, Cbz), 5,72 – 5,39 (m, 3H, CH=CH and NH), 5,03 (m, 3H, CH_2_ and NH), 4,80 (m, 1H, Hα), 4,17 (m, 1H, Hα), 3,67 (s, 3H, CH_3_), 2,52 – 2,27 (m, 2H, CH_2_), 1,36 (s, O^tBu^ and Boc). MS (ES^+^): m/z 493,3 (M+H)^+^.

1-Methyl 7-tert-butyl (2S,6R)-N^€^-(tert-butoxycarbonyl)-2,6-diaminopimelate (5). A solution of the unsaturated cross-product 4 (336 mg, 0.68 mmol) in ethyl acetate containing 10% wt% of Pd/C (10%) was hydrogenated at 30 ºC for 4-6 h under atmospheric pressure using a reaction balloon filled with hydrogen gas and a glass flask as the reaction vessel. The Pd/C was filtered through Whatman® filter paper 42 and the solvent was removed under reduced pressure to give 234 mg (quant) of the title compound as an amorphous white solid. ^1^H NMR (400 MHz, Chloroform-d) δ 7.95 (s, 1H, NH), 5.01 (d, J = 8.4 Hz, 1H, NH), 4.09 (m, 1H, Hα), 3.65 (s, 3H, CH_3_), 3.38 (dd, J = 7.3, 5.4 Hz, 1H, Hα), 1.82 – 1.62 (m, 5H, CH_2_), 1.60 – 1.44 (m, 1H, CH_2_), 1.38 (s, OtBu and Boc). MS (ES^+^): m/z 361.23 (M+H)^+^.

1-Methyl 7-tert-butyl (2S,6R)-N^α^-(N-fluoren-9-ylmethoxycarbonyl-D-glutamyl α-tert-butyl ester)-N^€^-(tert-butoxycarbonyl)-2,6-diaminopimelate (6). To a solution containing the properly protected meso-DAP intermediate 5 (132 mg, 0.36 mmol) in DMF (10 mL), Fmoc-(R)-Glu-OtBu (187 mg, 0.43 mmol), HATU (182 mg, 0.43 mmol) and DIPEA (142 μL, 0.81 mmol) were added. The reaction mixture was stirred at 30 ºC overnight and then evaporated to dryness. The residue was dissolved in dichloromethane (20 mL) and washed successively with aqueous solutions of citric acid (10%) (3 x 20 mL), saturated NaHCO_3_ (3 x 20 mL), and brine (3 x 20 mL). The organic phase was dried over anhydrous Na_2_SO_4_, filtered and evaporated to dryness. The residue was purified on preparative centrifugal circular thin layer chromatography (CCTLC) with Hex:EtOAc (6:4 to 1:1) as eluent, to afford 191 mg (67%) of compound 6 as a colorless syrup. HPLC [gradient: A:B, 50-100% of A in 10 min]: 7.7 min.^1^H NMR (400 MHz, Chloroform-d) δ 7.70 (d, J = 7.6 Hz, 2H, Fmoc), 7.54 (d, J = 7.5 Hz, 2H, Fmoc), 7.33 (t, 2H, Fmoc), 7.25 (t, 2H, Fmoc), 6.62 (d, J = 7.8 Hz, 1H, NH), 5.56 (d, J = 8.1 Hz, 1H, NH), 4.99 (d, J = 8.2 Hz, 1H, NH), 4.49 (m, 1H, Hα), 4.34 (m, 3H, Hα and CH_2_Fmoc), 4.15 (s, 1H, Hα), 4.06 (s, 1H, Hα), 3.65 (s, 3H, OMe), 2.22 (m, 3H, CH_2_), 1.80 (m, 2H, CH_2_), 1.67 (m, 2H, CH_2_), 1.55 (m, 3H, CH_2_), 1.37 (s, 27H, Boc and OtBu). MS (ES^+^): m/z 761.40 (M+H)^+^.

1-Methyl 7-tert-butyl (2S,6R)-N^α^-[N-(N-tert-butoxycarbonyl-L-alanyl)-D-glutamyl α-tert-butyl ester]-N^€^-(tert-butoxycarbonyl)-2,6-diaminopimelate (7). Intermediate 6 (191 mg, 0.24 mmol) was dissolved in a piperidine:DMF solution (20%). The reaction was stirring for 4 hours at room temperature. The solvent was removed under reduced pressure to give 135 mg (quant) of the corresponding free amine that was used directly to the next step. A solution of this amine (135 mg, 0.24 mmol) in DMF (10 mL), was treated with Boc-L-Ala-OH (56.1 mg, 0.29 mmol), HATU (122.8 mg, 0.29 mmol) and DIPEA (95 μL, 0.54 mmol). The reaction mixture was stirred at 30 ºC overnight and then evaporated to dryness. The residue was dissolved in dichloromethane (20 mL) and washed successively with aqueous solutions of citric acid (10%) (3 x 20 mL), saturated NaHCO_3_ (3 x 20 mL), and brine (3 x 20 mL). The organic phase was dried over anhydrous Na_2_SO_4_, filtered and evaporated to dryness. The residue was purified on preparative centrifugal circular thin layer chromatography (CCTLC) using dichloromethane:MeOH (100 to 10:1) as eluent, to afford 120 mg (67%) of compound 7 as a yellow foam. ^1^H NMR (400 MHz, Chloroform-d) δ 7.00 (s, 1H, NH), 5.03 (d, J = 8.2 Hz, 1H, NH), 4.94 (s, 1H, NH), 4.45 (m, 1H, Hα), 4.33 (s, 2H, NH and Hα), 4.09 (d, J = 9.1 Hz, 2H, Hα), 3.67 (s, 3H, OMe), 1.97 – 1.61 (m, 4H, CH_2_), 1.38 (s, 42H, Boc, OtBu and CH_2_), 1.31 (d, J = 7.1 Hz, 3H, CH_3_). MS (ES+): m/z 718,42 (M+H)^+^.

1-Carboxy 7-tert-butyl (2S,6R)-N^α^-[N-(N-tert-butoxycarbonyl-L-alanyl)-D-glutamyl α-tert-butyl ester]-N^€^-(tert-butoxycarbonyl)-2,6-diaminopimelate (8). To a solution at 0 ºC containing the methyl ester intermediate 7 (84 mg, 0.11 mmol) in THF (10 mL), a second solution of LiOH·H_2_O (4.95 mg, 0.11 mmol) in water (2.5 mL) was added. The resulting mixture was stirred at room temperature overnight. Then, an aqueous solution of hydrochloric acid (1N) was added to reach pH = 3-4, and the mixture was evaporated to dryness. The residue was dissolved in isobutanol (20 mL) and washed with H_2_O (3 x 20 mL). The organic phase was dried over anhydrous Na_2_SO_4_, filtered and evaporated to dryness to afford 56.8 mg (68%) of 8 as a yellow syrup. HPLC [gradient: A:B, 50-100% of A in 10 min]: 6.55 min. ^1^H NMR (400 MHz, DMSO-d_6_) δ 8.08 (d, J = 7.8 Hz, 1H, NH), 8.02 (d, J = 7.9 Hz, 1H, NH), 7.05 (d, J = 7.7 Hz, 1H, NH), 6.81 (d, J = 7.8 Hz, 1H, NH), 4.26 – 3.86 (m, 3H, Hα), 3.74 (m, 1H, Hα), 2.17 (dd, J = 13.3, 5.5 Hz, 2H, CH_2_), 1.98 – 1.71 (m, 3H), 1.66-1.36 (m and s, 41H, Boc, OtBu and CH_2_), 1.18 (d, J = 7.1 Hz, 3H, CH_3_). MS (ES+): m/z 703,41 (M+H)^+^.

1-(D-alanyl tert-butylester) 7-tert-butyl (2S,6R)-N^α^-[N-(N-tert-butoxycarbonyl-L-alanyl)-D-glutamyl α-tert-butyl ester]-N^€^-(tert-butoxycarbonyl)-2,6-diaminopimelate (9). To a solution containing the acid intermediate 8 (68.5 mg, 0.097 mmol) in DMF (10 mL), H-D-Ala-OtBu (21.2 mg, 0.12 mmol), HATU (40.3 mg, 0.09 mmol) and DIPEA (39 μL, 0.19 mmol) were added. The reaction mixture was stirred at 30 ºC overnight and then evaporated to dryness. The residue was dissolved in dichloromethane (20 mL) and washed successively with aqueous solutions of citric acid (10%) (3 x 20 mL), saturated NaHCO_3_ (3 x 20 mL), and brine (3 x 20 mL). The organic phase was dried over anhydrous Na_2_SO_4_, filtered and evaporated to dryness. The residue was purified on preparative centrifugal circular thin layer chromatography (CCTLC) using dichloromethane:MeOH (100:1 to 20:1) as eluent, to afford 55 mg (68%) of compound 9. EM (ESI^+^): m/z 830.5 (M+H)^+^. ^1^H NMR (400 MHz, Acetone-d_6_) δ 7.67 (d, J = 7.5 Hz, 1H, NH), 7.53 (d, J = 8.4 Hz, 1H, NH), 7.35 (d, J = 7.6 Hz, 1H, NH), 6.27 (s, 1H, NH), 5.94 (d, J = 8.3 Hz, 1H, NH), 4.19 (m, 3H, Hα), 4.01 (m, 1H, Hα), 3.84 (m, 1H, Hα), 2.19 (m, 4H, CH_2_), 1.80-1.45 (m, 4H, CH_2_), 1.42 – 1.27 (m, 47H, Boc, OtBu and CH_2_), 1.23 (2d, J = 2.33 Hz, 3H, CH_3_), 1.22 (d, J = 2.46 Hz, 3H, CH_3_).

(S)-Alanyl-γ-(R)-glutamyl-meso-diaminopimelyl-(R)-alanine (PGN-1). To a solution of the protected intermediate 9 (55 mg, 0.06 mmol) in CH_2_Cl_2_ (10 mL), TFA was added. After stirring at room temperature overnight, the solution was evaporated to dryness and co-evaporated several times with CH_2_Cl_2_ and MeOH to remove residual TFA. ^1^H NMR (500 MHz, Methanol-d_4_) δ 4.40 (m, 3H, Hα), 3.99 (m, 1H, Hα), 3.87 (m, 1H, Hα), 2.37 (m, 2H, CH_2Glu_), 2.27 (m, 1H, CH_Glu_), 1.96 (m, 2H, CH_DAP_, CH_Glu_), 1.86 (m, 2H, CH_DAP_, CH_DAP_), 1.68 (m, 1H, CH_DAP_), 1.53 (m, 5H, CH_3(S)Ala_ and CH_2_CH_2DAP_CH_2_), 1.39 (d, 3H, CH_3(R)Ala_). ^13^C NMR (126 MHz, Methanol-d_4_) δ 174.52, 173.19, 173.16, 172.27, 169.82, 168.23, 52.84, 52.80, 51.94, 48.87, 48.07, 31.26, 31.23, 29.83, 27.15, 21.09, 16.41. To obtain the hydrochloride salt, a solution of the TFA salt form in water was treated with several drops of hydrochloric acid (37%). The aqueous solution was washed several times with ethyl acetate and then lyophilized to afford 11.7 mg (35%) of PGN-1 as a green foam. HRMS (ESI, positive): m/z calculated for C_18_H_32_N_5_O_9_ 462.2267; found 462.2205. This is the form in which the compound was tested.

1-[(R)-(1-tert-butoxypropan-2-yl)amino] 7-tert-butyl (2S,6R)-N^α^-[N-(N-tert-butoxycarbonyl-L-alanyl)-D-glutamyl α-tert-butyl ester]-N^€^-(tert-butoxycarbonyl)-2,6-diaminopimelate (10). To a solution containing the acid intermediate 8 (40 mg, 0.057 mmol) in DMF (10 mL), tert-butyl-D-alaninol (9 mg, 0.068 mmol), HATU (23.5 mg, 0.057 mmol) and DIPEA (19 μL, 0.11 mmol) were added. The reaction mixture was stirred at 30 ºC overnight and then evaporated to dryness. The residue was dissolved in dichloromethane (20 mL) and washed successively with aqueous solutions of citric acid (10%) (3 x 20 mL), saturated NaHCO_3_ (3 x 20 mL), and brine (3 x 20 mL). The organic phase was dried over anhydrous Na_2_SO_4_, filtered and evaporated to dryness. The residue was purified on preparative centrifugal circular thin layer chromatography (CCTLC) using dichloromethane:MeOH (100:1 to 10:1) as eluent, to afford 32.8 mg (60%) of compound 10 as a colorless syrup. HPLC [gradient: A:B, 50-100% of A in 10 min]: 6.45 min. ^1^H NMR (400 MHz, Acetone-d_6_) δ 7.60 (d, J = 8.5 Hz, 1H, NH), 7.26 (d, J = 7.8 Hz, 2H, NH), 6.32 (s, 1H, NH), 5.99 (d, J = 8.3 Hz, 1H, NH), 4.29 (m, 2H, 2Hα), 4.13 (m, 1H, Hα), 3.98 (m, 2H, 2Hα), 3.35 (m, 1H, CH_2_OH), 3.24 (m, 1H, CH_2_OH), 2.26 (m, 1H, CH_2_), 1.91 – 1.56 (m, 2H, CH_2_), 1.42 (d, J = 13.1 Hz, 43H, Boc, OtBu and CH_2 DAP_), 1.36 (d, J = 7.2 Hz, CH_3_), 1.15 (m, 12H, O^t^Bu and CH_3_). MS (ES+): m/z 817,05 (M+H)^+^.

1-[(R)-(1-hydroxypropan-2-yl)amino] (2S,6R)-N^α^-[N-(L-alanyl)-D-glutamyl]-2,6-diaminopimelate (PGN-2). To a solution of the protected intermediate 10 (32.8 mg, 0.04 mmol) in CH_2_Cl_2_ (10 mL), TFA was added. After stirring at room temperature overnight the solution was evaporated to dryness and co-evaporated several times with dichloromethane and MeOH to remove residual TFA. To obtain the hydrochloride salt a solution of the TFA salt form in water was treated with several drops of hydrochloric acid (37%). The aqueous solution was washed several times with ethyl acetate and then lyophilized to afford 19.1 mg (69%) of PGN-2 as a green foam. This is the form in which the compound was characterized and tested. HRMS (ESI, positive): m/z calculated for C_18_H_35_N_5_O_8_ 449.2486; found 449.2481. ^1^H NMR (500 MHz, D_2_O) δ 4.24 (m, 1H, Hα), 4.10 (m, 1H, Hα), 3.98 (q, J = 7.0 Hz, 1H, Hα), 3.85 (m, 1H, CH, Hα), 3.80 (m, 1H, CHCH_2_OH), 3.41 (m, 1H, CH_2A_OH), 3.35 (m, 1H, CH_2B_OH), 2.26 (m, 2H, CH_2Glu_), 2.07 (m, 1H, CH_Glu_), 1.88 (m, 1H, CH_Glu_), 1.80 (m, 2H, CH_2DAP_), 1.63 (m, 2H, CH_2DAP_), 1.41 (d, J = 7.0 Hz, 3H, CH_3(S)Ala_), 1.35 (m, 2H, CH_2_CH_2DAP_CH_2_), 0.96 (d, J = 6.8 Hz, 3H, CH_3_CHCH_2_OH). ^13^C NMR (125 MHz, D_2_O) δ 174.76, 174.65, 173.27, 172.33, 170.74, 64.22, 53.70, 52.90, 52.14, 48.97, 47.21, 31.21, 30.60, 29.34, 26.40, 20.76, 16.51, 15.71.

1-(1-tert-butoxy-2-hydroxypropylamino) 7-tert-butyl (2S,6R)-N^α^-[N-(N-tert-butoxycarbonyl-L-alanyl)-D-glutamyl α-tert-butyl ester]-N^€^-(tert-butoxycarbonyl)-2,6-diaminopimelate (11). To a solution containing the acid intermediate 8 (56.8 mg, 0.081 mmol) in DMF (10 mL), tert-butyl-1-amino-2-propanol (12.7 mg, 0.09 mmol), HATU (33.4 mg, 0.081 mmol) and DIPEA (14 μL, 0.081 mmol) were added. The reaction mixture was stirred at 30 ºC overnight and then evaporated to dryness. The residue was dissolved in dichloromethane (20 mL) and washed successively with aqueous solutions of citric acid (10%) (3 x 20 mL), saturated NaHCO_3_ (3 x 20 mL), and brine (3 x 20 mL). The organic phase was dried over anhydrous Na_2_SO_4_, filtered and evaporated to dryness. The residue was purified on preparative centrifugal circular thin layer chromatography (CCTLC) using dichloromethane:MeOH (100:1 to 10:1) as eluent, to afford 43 mg (65%) of compound 11 as a colorless oil. ^1^H NMR (400 MHz, Acetone-d_6_) δ 7.51 (s, 1H, NH), 7.38 (d, J = 19.5 Hz, 1H, NH), 7.20 (dd, J = 17.8, 7.7 Hz, 1H, NH), 6.23 (s, 1H, NH), 5.86 (d, J = 8.3 Hz, 1H, NH), 4.19 (m, 2H, Hα), 4.01 (s, 1H, Hα), 3.84 (m, 1H, Hα), 3.67 (m, 1H, Hα), 2.99 (m, 2H, CH_2_), 2.17 (m, 5H, CH_2_), 1.78 – 1.56 (m, 2H, CH_2_), 1.51 (m, 2H, CH_2_), 1.32 (m, 37H, OtBu, Boc and CH_2_), 1.04 (s, 12H, OtBu and CH_3_), 0.92 (t, J = 5.5 Hz, 3H, CH_3_).

1-[2-hydroxypropylamino] (2S,6R)-N^α^-[N-(L-alanyl)-D-glutamyl]-2,6-diaminopimelate (PGN-3). To a solution containing the protected intermediate 11 (43 mg, 0.05 mmol) in dichloromethane (10 mL), TFA was added. After stirring at room temperature overnight the solution was evaporated to dryness and the solution was evaporated to dryness and co-evaporated several times with dichloromethane and MeOH to remove residual TFA. To obtain the hydrochloride salt a solution of the TFA salt form in water was treated with several drops of hydrochloric acid (37%). The aqueous solution was washed several times with ethyl acetate and then lyophilized to afford 22.1 mg (63%) of PGN-3 as a green foam. This is the form in which the compound was characterized and tested. HRMS (ESI, positive): m/z calculated for C_18_H_35_N_5_O_8_ 449.2486; found 449.2499.^1^H NMR (500 MHz, D_2_O) δ 4.11 (m, 2H, 2Hα), 3.98 (q, J = 7.0 Hz, 1H, Hα), 3.78 (q, J = 6.1 Hz, 1H, CH_3_CHOH), 3.62 (m, 1H, Hα), 3.09 (m, 2H, CH_2_N), 2.25 (m, 2H, CH_2 Glu_), 2.02 (m, 1H, CH_Glu_), 1.90 – 1.48 (m, 4H, CH_Glu,_ CH_2DAP_ and CH_2DAP_), 1.41 (d, J = 7.0 Hz, 3H, CH_3(S)Ala_), 1.33 (m, 2H, CH_2_CH_2DAP_CH_2_), 1.01 (d, J = 6.2 Hz, 3H, CH_3_CHOH). ^13^C NMR (100 MHz, D_2_O) δ 175.43, 174.23, 170.47, 66.14, 54.51, 53.91, 49.19, 46.00, 31.76, 30.67, 29.89, 27.20, 20.98, 19.43, 16.61.

**References**

1. Chowdhury, A.R.; Boons G-J. The synthesis of diaminopimelic acid containing peptidoglycan fragments using metathesis cross coupling, Tetrahedron lett. 2005, 46, 1675-1678.
2. Roychowdhury, A.; Wolfert, M.A.; Boons G-J. Synthesis and proinflammatory properties of muramyl tripeptides containing lysine and diaminopimelic acid moieties. Chem BioChem 2005, 6, 2088-2097.
3. Gao, Y.; Lane-Bell, P.; Vederas, J.C. Stereoselective synthesis of meso-2,6-diaminopimelic acid and its selectively protected derivatives. J.Org. Chem. 1998, 63, 2133-2143.
4. Kawasaki, A.; Karasudani, Y.; Otsuka, Y.; Hasegawa, M.; Inohara, N.; Fujimoto, Y; Fukase, K. Synthesis of diaminopimelic acid containing peptidoglycan fragments and tracheal cytotoxin (TCT) and investigation of their biological functions. Chemistry 2008, 14, 10318-10330.
5. Saito, Y.; Yoshimura, H.; Wakamatsu, H.; Takahata, H. A facile synthesis of fully protected meso-diaminopimelic acid (DAP) and its application to the preparation of lipophilic N-acyl iE-DAO. Molecules 2013, 18, 1162-1173.
6. Paradisi, F.; Porzi, G.; Rinaldi, S.; Sandri, S. A simple asymmetric synthesis of (+)- and (-)-2,6-diaminopimelic acids, Tetrahedron Asymmetry 2000, 11, 1259-1262.
7. Paradisi, F.; Piccinelli, F.; Porzi, G.; Sandri, S. Enantioselective synthesis of 2,6-diaminopimelic acid derivatives, Tetrahedron Asymmetry 2002, 13, 497-502.
8. Collier, P.N.; Patel, I.; Taylor, R.J.K. A concise, stereoselective synthesis of meso-2,6-diaminopimelic acid. Tetrahedron Lett. 2001, 42, 5953-5954.
9. Nolen E.G.; Fedorka, C.J.; Blicher, B. Synthesis of orthogonally protected S,S-2,6-diaminopimelic acid via olefin cross-metathesis. Synth. Commun. 2006, 36, 1707-1713.
10. Miller, S. J.; Blackwell, H. E.; Grubbs, R. H. Application of ring-closing metathesis to the synthesis of rigidified amino acids and peptides. J. Am. Chem. Soc. 1996, 118, 9606-9614.
11. Armstrong, A.; Brackenridge, I.; Jackson, R.F.W.; Kirk, J.M. A new method for the preparation of tertiary butyl ethers and esters. Tetrahedron Lett. 1988, 29, 2483-2486.
12. Rothman D. M.; Vazquez, M. E.; Vogel, E. M.; Imperiali, B. Caged phospho-amino acid building blocks for solid-phase peptide synthesis. J. Org.Chem., 2003, 68, 6795-6798.
13. Jakopin, Ž.; Gobec, M.; Kodela, J.; Hazdovac, T.; Mlinarič-Raščan, I.; Dolenc, M. S. Synthesis of conformationally constrained γ-D-glutamyl-meso-diaminopimelic acid derivatives as ligands of nucleotide-binding oligomerization domain protein 1 (Nod1). European J. Med. Chem. 2013, 69, 232-243.

- **^1^HNMR, ^13^CNMR and mass spectra of PGN-1, PGN-2 and PGN-3**

**PGN-1**

**PGN-2**

**PGN-3**
