# Supplementary material for: Peptidoglycan editing in non-proliferating intracellular Salmonella as source of interference with immune signaling
Source: PLoS Pathog. 2022 Jan 25;18(1):e1010241. doi: 10.1371/journal.ppat.1010241 (PMC8815878; doi:10.1371/journal.ppat.1010241)
Supplement: S1 Table — (DOCX) [file ppat.1010241.s009.docx]

**S1 Table.** *m/z* values obtained in the untargeted MS/MS comparative analysis that include candidates to correspond to Intracellular *Salmonella* Muropeptides (ISM) (see also S2 Fig).

| **Muropeptide**  ***m/z*** | **RT (min)** | **Intracellular-specific** |
| --- | --- | --- |
| 840.4 | 2.3 | Yes |
| 969.4 | 2.9 | Yes |
| 928.4 | 4.7 | Yes |
| 632.3 | 4.7 | No |
| 590.1 | 4.8 | No |
| 622.3 | 4.9 | Yes |
| 520.1 | 5.0 | No |
| 646.8 | 5.1 | No |
| 722.4 | 5.4 | No |
| 708.9 | 5.6 | No |
| 483.7 | 5.8 | No |
| 699.3 | 5.9 | No |
| 851.4 | 6.6 | No |
| 890.9 | 6.8 | Yes |
| 926.4 | 7.4 | Yes |
| 893.4 | 7.7 | No |
| 933.4 | 8.5 | No |
| 960.5 | 9.9 | No |
