# Supplementary material for: Peptidoglycan editing in non-proliferating intracellular Salmonella as source of interference with immune signaling
Source: PLoS Pathog. 2022 Jan 25;18(1):e1010241. doi: 10.1371/journal.ppat.1010241 (PMC8815878; doi:10.1371/journal.ppat.1010241)
Supplement: S2 Table — (DOCX) [file ppat.1010241.s010.docx]

**S2 Table.** Most abundant muropeptides detected by untargeted MS/MS in the PG samples of extracellular and intracellular *S.* Typhimurium

| Muropeptide | Area (%) ^&^ | | |
| --- | --- | --- | --- |
|  | **Extracellular** | **Intracellular ^¶^** | |
|  | **LB** | **NRK-49F** | **BJ-5ta** |
| ISM-1 * | 0.00 | 0.29 | 0.06 |
| ISM-2 * | 0.00 | 0.06 | 0.09 |
| M3 | 7.57 | 6.17 | 3.61 |
| M4G | 0.94 | 0.12 | 0.16 |
| M3G | 2.49 | 0.38 | 0.11 |
| ISM-3 * | 0.01 | 1.38 | 1.47 |
| M4 | 39.40 | 47.31 | 48.99 |
| M2 | 5.69 | 2.14 | 3.28 |
| ISM-4 * | 0.00 | 0.61 | 0.62 |
| D33-Gly | 0.64 | 0.27 | 0.15 |
| M3-Lpp | 1.38 | 1.80 | 0.80 |
| D33 | 1.23 | 0.76 | 0.85 |
| D44-Gly | 1.30 | 0.26 | 0.62 |
| D43-Gly | 2.24 | 0.69 | 0.34 |
| D43 | 3.44 | 1.91 | 2.47 |
| ISM-5 * | 0.00 | 0.21 | 0.22 |
| D34 | 4.06 | 6.28 | 5.62 |
| ISM-6 * | 0.00 | 0.51 | 0.63 |
| D44 | 21.65 | 21.22 | 22.15 |
| M44-Lactyl | 0.18 | 0.02 | 0.03 |
| M4N | 0.57 | 1.01 | 0.79 |
| D43-Lpp | 3.10 | 2.02 | 2.00 |
| T443 | 0.56 | 0.73 | 0.71 |
| T444 | 1.71 | 1.62 | 1.91 |
| D44N | 1.82 | 2.26 | 2.32 |

* Muropeptides enriched in the PG of intracellular *S.* Typhimurium, named as ISM for “Intracellular *Salmonella* Muropeptides” (see Fig 1).

^&^ The relative amount of each muropeptide was calculated integrating the area of the corresponding extracted ion chromatogram from the MS data. Values are represented as the percentage of the total area. Muropeptide structures are shown in S1C Fig.

**^¶^** The samples of intracellular bacteria were collected at 24 h post infection.
