# Supplementary material for: Peptidoglycan editing in non-proliferating intracellular Salmonella as source of interference with immune signaling
Source: PLoS Pathog. 2022 Jan 25;18(1):e1010241. doi: 10.1371/journal.ppat.1010241 (PMC8815878; doi:10.1371/journal.ppat.1010241)
Supplement: S3 Table — (DOCX) [file ppat.1010241.s011.docx]

**S3 Table.** *S.* Typhimurium strains and plasmids used in this study

| S. Typhimurium strain | Genotype | Source/  reference |
| --- | --- | --- |
| SV5015 | SL1344 *hisG^+^* | [1] |
| MD1120 | SV5015 *phoP7953*::Tn*10* | [2] |
| MD3238 | SV5015 *dacC*::3xFLAG-Km | This study |
| MD3790 | SV5015 *dacD*::3xFLAG *mltF*::3xFLAG *mepA*::3xFLAG *mltB*::3xFLAG-Km | [3] |
| MD5225 | SV5015 *ynhG*::3xFLAG | This study |
| MD2562 | SV5015 ∆*dacC* ∆*dacD* | This study |
| MD3952 | SV5015 ∆*amiA* ∆*amiC*::Km | This study |
| MD3964 | SV5015 ∆*amiB* ∆*amiC*::Km | This study |
| MD5283 | SV5015 ∆*ycbB* ∆*ynhG* | This study |
| Plasmids | **Genotype** | **Source/reference** |
| pKD4 | Km^R^, Amp^R^ | [4] |
| pKD13 | Km^R^, Amp^R^ | [4] |
| pKD46 | *ϒ*, *β*, exo. Amp^R^ | [4] |
| pSUB11 | 3xFLAG sequence, Km^R^ | [4] |
| pCP20 | *FLP*^+^, Amp^R^, Cm^R^ | [5] |

**References**

1. Vivero A, Baños RC, Mariscotti JF, Oliveros JC, García-del Portillo F, Juárez A, et al. Modulation of horizontally acquired genes by the Hha-YdgT proteins in Salmonella enterica serovar Typhimurium. J Bacteriol. 2008;190: 1152–1156. doi:10.1128/JB.01206-07

2. Núñez-Hernández C, Tierrez A, Ortega AD, Pucciarelli MG, Godoy M, Eisman B, et al. Genome expression analysis of nonproliferating intracellular Salmonella enterica serovar Typhimurium unravels an acid pH-dependent PhoP-PhoQ response essential for dormancy. Infect Immun. 2013;81: 154–165. doi:10.1128/IAI.01080-12

3. Cestero JJ, Castanheira S, Pucciarelli MG, García-Del Portillo F. A Novel Salmonella Periplasmic Protein Controlling Cell Wall Homeostasis and Virulence. Front Microbiol. 2021;12: 633701. doi:10.3389/fmicb.2021.633701

4. Datsenko KA, Wanner BL. One-step inactivation of chromosomal genes in Escherichia coli K-12 using PCR products. Proc Natl Acad Sci U S A. 2000;97: 6640–6645. doi:10.1073/pnas.120163297

5. Cherepanov PP, Wackernagel W. Gene disruption in Escherichia coli: TcR and KmR cassettes with the option of Flp-catalyzed excision of the antibiotic-resistance determinant. Gene. 1995;158: 9–14. doi:10.1016/0378-1119(95)00193-a
