# Supplementary material for: Peptidoglycan editing in non-proliferating intracellular Salmonella as source of interference with immune signaling
Source: PLoS Pathog. 2022 Jan 25;18(1):e1010241. doi: 10.1371/journal.ppat.1010241 (PMC8815878; doi:10.1371/journal.ppat.1010241)
Supplement: S4 Table — (DOCX) [file ppat.1010241.s012.docx]

**S4 Table.** Oligonucleotides used in this study

| **Oligonucleotide** | **Sequence (5’ to 3’)*** |
| --- | --- |
|  |  |
| FLAG-DacC Fw | GCTGATGAAACTCCATCAGTGGTTTGGCAGTTGGTTCTCGGACTACAAAGACCATGACGG |
| FLAG-DacC Rv | CCGTAGCCGGATGCGACGCGCACCCGGCTACGGAGTTATTCATATGAATATCCTCCTTAG |
| FLAG‐YnhG Fw | CCGCTCAGAACGGTTTTGTCGGGGAAGAGGGGCAAACGCGCGCGACGCAGGACTACAAAGACCATGACGG |
| FLAG‐YnhG Rv | AAATGGCGCACATCGTGCGCCATTTTTTTGTCCGTCGTTTGCTGCAAAGGCATATGAATATCCTCCTTAG |
| KO‐PBP 6 Fw | TTACACGGTGGTGTGACGTGTGTTTTTGGAATGGATACTCGGGTGGTACTGTGTAGGCTGGAGCTGCTTC |
| KO‐PBP 6 Rv | TAACCGCCCGTAGCCGGATGCGACGCGCACCCGGCTACGGAGTTATTTTAATTCCGGGGATCCGTCGACC |
| KO‐PBP 6b Fw | CCCTTTTGACACCTCAGATGACGGTGAACGGTGTGTGTGACAACGGCTTAGTGTAGGCTGGAGCTGCTTC |
| KO‐PBP 6b Rv | TTAACTGAACTTCCGTAAAAAGAACGGCAAATAGAGACCATCCTGAGGACATTCCGGGGATCCGTCGACC |
| KO‐AmiA Fw | TACGCGATACGATATAACATCTTGAACTTAATTTTCACAACTCAGGCCGTGTGTAGGCTGGAGCTGCTTC |
| KO‐AmiA Rv | CTGCAGGCGTAGCAGAAACTGTTTTACGTGATGTGCATCGGGTTTCATGACATATGAATATCCTCCTTAGT |
| KO‐AmiB Fw | GGGTCATTCGTTACTGGCGCGTTTAGCCGATTAGCTATAAAGGTGGCGGGGTGTAGGCTGGAGCTGCTTC |
| KO‐AmiB Rv | ATTTGGTTCGCAAGCTGCGGCGGCAGAACCTGAATCGGCATGAAATCTCCCATATGAATATCCTCCTTAGT |
| KO‐AmiC Fw | TGCGGGCAGCATCCTACTTACCCGCGCAATAAACTCGCCGTCATCTCAGGGTGTAGGCTGGAGCTGCTTC |
| KO‐AmiC Rv | ATTTGGATGAACTTTGTATGATCTCTATTTAGTTTTTGCTCGGGAGAAGCATTCCGGGGATCCGTCGACC |
| KO‐YcbB Fw | GCGGGCAGCGCAGTCATGCGGAGTATGATAACGAAAACAGGGGGCAAGGGGTGTAGGCTGGAGCTGCTTC |
| KO‐YcbB Rv | CCCCGTTGAAGCCAAACATTAGGACAACATATTTACCAGAACTTCTTCATATTCCGGGGATCCGTCGACC |
| KO‐YnhG Fw | GCGCTACCAGTCGTCGCAGAGGGCATCACCAGGAATACAGGAGGTTTGGTGTGTAGGCTGGAGCTGCTTC |
| KO‐YnhG Rv | AAATGGCGCACATCGTGCGCCATTTTTTTGTCCGTCGTTTGCTGCAAAGGATTCCGGGGATCCGTCGACC |
| KO‐STM1940 Fw | CGCGCACGGTTGCCTTAATCATGTCTTCCGCGATAACACAGTTTGTCAACGTGTAGGCTGGAGCTGCTTC |
| KO‐STM1940 Rv | AATGGCGGTTTTGGTCAGTCTCTATATTATGCTTTCCCCGCTTTACGGAGATTCCGGGGATCCGTCGACC |
| KO‐NlpC Fw | AATTTCGACGCTAAATTAATACCAAAATAAAAACAGAGGATTGTTGCGGCGTGTAGGCTGGAGCTGCTTC |
| KO‐NlpC Rv | AATGGCGGTTTTGGTCAGTCTCTATATTATGCTTTCCCCGCTTTACGGAGATTCCGGGGATCCGTCGACC |
| YnhA Fw | CCAACGGCATTATCGAATTGCA |
| YnhA Rv | CTAAGAGTGGCGGCCTTAGC |
| LppB Fw | GTTGTCCAGACGTTGGTTCG |
| LppB Rv | GTCTTCCGATGTACAGACGCTGAG |
| DeoR Fw | CGCAGCGCCAGTCATTATCT |
| DeoR Rv | ACGTTTCATGGAAATCAAGCG |
| YliJ Fw | AATTCGGCGTTAACCAGGATG |
| YliJ Rv | TCGATACCGGCTTCAATTGC |
| SbmC Fw | ACACCGTGAAGCAGGGTTTT |
| SbmC Rv | TGATAAGCGCTATCCTGCTCG |
| PhsC Fw | GGTGCTGCTCATGCTTGTTG |
| PhsC Rv | CCGATAGTGACGCCCGTTAC |
| STM2449 Fw | TTTTCGCCAGGAAGATTTCG |
| STM2449 Rv | AATTTGGATTTTCGGACACCC |
| HemF Fw | CAGTTTCTGCTACGCCTGCA |
| HemF Rv | GCACGTTAGCATGGCTGGT |
| YjeE Fw | TGGCGCGACCGTTATTTATC |
| YjeE Rv | GCGCCTGGTAATCAATATGGA |
| MutL Fw | CAGCTTGCGAACCAAATCG |
| MutL Rv | TTAGCGTCAAACGCGAGACC |
| ArgA Fw | AGCCTCGGTATTCGCCTTGT |
| ArgA Rv | GGATACGGCCGCTATGACAG |
| MukB Fw | GAGCAGGTTGTGACCGCGAA |
| MukB Rv | TCGGATCTTCCGACAGACGCAA |
| YcbK Fw | TAATCGCCGCAAGCTGCTGG |
| YcbK Rv | TTATTCGCGCGATAGTCACGGA |
| STM1939 Fw | TTCGTCTACCGCTTCTCTGTCA |
| STM1939 Rv | GAACCGCAACCGCTATACACA |
| STM1941 FW | CAATACATACTGTAGCGCGTCAGTT |
| STM1941 Rv | TGAGCCTGAGAGTGCAATCG |
| BtuD Fw | CCTTGCTGCCGTTGTACTAC |
| BtuD Rv | TGTGGTTCAGATCGTGGCTA |
| YdiV Fw | GAAAACGCTCGGCTTGTT |
| YdiV Rv | AAGTCACTAATTGCAGGCGG |

(*) Blue colour, sequence of the pSUB11 vector
