# Supplementary material for: Peptidoglycan editing in non-proliferating intracellular Salmonella as source of interference with immune signaling
Source: PLoS Pathog. 2022 Jan 25;18(1):e1010241. doi: 10.1371/journal.ppat.1010241 (PMC8815878; doi:10.1371/journal.ppat.1010241)
Supplement: Attachment — Submitted filename: Point-to-point-responses-PPATHOGENS-D-21-01404.pdf [file ppat.1010241.s013.pdf]

Point-to-point responses to reviewers of previous submission PPATHOGENS-D-21-01404

Reviewer #1

In their manuscript 'Peptidoglycan editing in non-proliferating intracellular Salmonella as source of interference with immune signaling' Hernandez et al. apply state-of-the-art chromatography techniques to reveal the intracellular spectrum of peptides that build the peptidoglycan.

The authors employ an established tissue culture infection model (fibroblasts) to mimick persistent (non-proliferating) S. enterica infection conditions, to compare peptidoglycan profiles of this intracellular population to a proliferating in vitro culture (LB).
This is a comprehensive study of general interest revealing a wealth of both known and novel muropeptide modifications in S. enterica that are likely to be relevant in other pathogens. The manuscript is carefully written and presented, and the study highlights the eminent role of muropeptide modifications for immune evasion during persistent intracellular infection of this important pathogen. The observed diversity in muropeptide species is remarkable and of great interest. The structural analysis of muropeptide species by UPLC coupled to untargeted MS/MS appears to be of high quality.

We thank the reviewer for the overall highly positive evaluation of our study.

A weakness of the study is that it remains largely descriptive and the implicated peptidoglycan-editing enzymes require more attention and additional experimentation to show their contribution to the observed peptidoglycan modifications.

We have tested *Salmonella* mutant strains defective in candidate enzymes for some of the modifications in PG described, specifically those involving the group of L,D-crosslinked muropeptides that we named ISM-1, ISM-2 and ISM-4 (see Fig. 2). These mutations include defects in enzymes as AmiA, AmiB, AmiC, DacC, DacD, LdtD(YcbB) and LdtE (YnhG). Mutants lacking these enzymes were used to infect reporter fibroblasts from knock-in mice expressing GFP-p65, and the rate of NF-κB nuclear translocation determined for each strain at early (60 min) and late (180 min) post infections (**new Fig. 6**). These times were set according to our previous observations of NF-kB nuclear translocation in S. Typhimurium-infected fibroblasts, which show an early stimulation phase followed by a marked attenuation of the immune response triggered by the pathogen (Ramos-Marques et al. Virulence, 2017, PMID: 27575017). The new data obtained did not reveal differences in the NF-κB response between wild-type and any of the mutants tested. However, as it is discussed in the new version of the manuscript, the new structural analyses performed in peptidoglycan isolated from these mutants in the intracellular infection demonstrate that **all these mutants are still capable of modifying the terminal D-alanine of a fraction of stem tetrapeptides to the amino alcohol alaninol when located inside the infected cell** (**new S6 Fig**.). Interestingly, the assays performed with synthetic tetrapeptides bearing this amino alcohol unequivocally show that this D-alanine to alaninol modification is sufficient to attenuate NF-κB nuclear translocation (**see Fig. 7**). We therefore conclude that this unprecedented D-alanine to alaninol modification might be central in the strategy used by non-proliferating intracellular *S.* Typhimurium to attenuate immune signaling. Please, note that the enzyme(s) involved in this D-alanine to alaninol modification are yet unknown, so we cannot approach the effect with whole bacteria given the impossibility of generating the corresponding mutant. We are currently pursuing this goal.

Reviewer #2:

Hernandez and colleagues present a study on the characterization of the peptidoglycan (PGN) composition of non-proliferating intracellular Salmonella and suggest that modifications of the PGN are associated with interference of the immune response. Technically, the study is well done but falls short of providing convincing evidence that the observed modifications are indeed real (lack of statistical analysis) and associated with reduced immune response.

This reviewer does not consider in his/her comment our previous publications in which we describe in detail the way in which these samples are prepared. The previous manuscript (PPATHOGENS-D-21-01404) cites in lines 380-382 reference 7 (Nunez-Hernandez et al., 2013) in which we describe that the material from nonproliferating intracellular *Salmonella* is obtained in series of four 500 cm2 flaks each in up to **20 independent experiments**. The bacterial pellets from each series of four flasks are pooled to finally recover the material by centrifugation. **In lines 394-395 we clearly state that for the experiments described in this study, a minimum of 140 500-cm2 flasks were used**. Note that the protocol involves lysis of the infected fibroblasts in a solution containing 1% phenol-19% ethanol (method originally described by Eriksson et al., 2003, PMID: 12492857), which means "instant lysis" before any further manipulation of the sample and, therefore, prevents any undesirable artifact. This is clearly explained in lines 389-398 of the manuscript, so we disagree with the reviewer in the lack of reproducibility and do not understand his/her reservation for the lack of statistical analyses. **The fact that samples are the result of pooling more than one hundred flasks automatically leads to reliable and solid data in statistical terms**.

Part II – Major Issues

Reviewer #1: • The experimental context for the peptidoglycan analysis is missing. In which LB growth phase were the bacteria analysed. How does the fibroblast infection look like in terms of CFU? The authors emphasize throughout the manuscript that the data are representative of non-proliferating intracellular bacteria, therefore it would be very helpful to show a simple CFU curve for the infection.

We consider these as minor comments. From our initial publication in 1998:

Inhibition of Salmonella intracellular proliferation by non-phagocytic eucaryotic cells.
Martínez-Moya M, de Pedro MA, Schwarz H, García-del Portillo F. Res Microbiol. 1998 May;149(5):309-18. doi: 10.1016/s0923-2508(98)80436-1. PMID: 9766231

We have repeatedly shown the progression of the infection in distinct fibroblasts from mouse, rat and human cell lines (PMID: 1155359, 12819049, 20368348). In all these publications we present data of intracellular proliferation rates with the corresponding curves.

Regarding the control sample in LB medium, bacteria were collected from an overnight culture in static (non-shaking) condition. Under this condition, bacteria are highly competent for invasion and, therefore, PG was isolated from bacteria as they were grown for infecting fibroblasts. This detail was missing in the manuscript and it is now included in the new submission. We thank the reviewer for pinpointing it.

• Related to the first point: it might be an overstatement that the detected PG modifications are relevant for/occurring only in non-proliferating bacteria. After all, the analysis was not done for proliferating intracellular bacteria. Perhaps, the authors should qualify these statements or (alternatively) analyse PG profiles of proliferating intracellular bacteria

The PG profile of proliferating intracellular *Salmonella* has been already analyzed in bacteria recovered from epithelial cells. Please, see our previous work in HeLa epithelial cells:

Peptidoglycan structure of Salmonella typhimurium growing within cultured mammalian cells.
Quintela JC, de Pedro MA, Zöllner P, Allmaier G, Garcia-del Portillo F. Mol Microbiol. 1997 Feb;23(4):693-704. doi: 10.1046/j.1365-2958.1997.2561621.x. PMID: 9157241

• L149-163: The authors suggest that EcgA might be involved in the production of the trimmed peptides. This enzyme has been pre-characterized by the same group and a knockout strain already exists. The peptide motifs released by this enzyme activity are expected to trigger immune responses. Therefore, please analyse the effect of an ecgA knockout on intracellular PG patterns and/or demonstrate an effect on NOD-dependent immune-responses. Alternatively, the authors could demonstrate a function for one of the other two enzymes, YnhG and PBP6b, that were upregulated during infection

Mutants lacking these enzymes have been generated and new conclusions have been reached. In the case of the L,D-transpeptidase LdtE(YnhG), we have generated a double mutant lacking the two L,D-transpeptidades known to form the DAP-DAP bridge, i.e. LdtD(YcbB) and LdtE(YnhG). The analysis of the PG of this mutant purified from extracellular and **intracellular** bacteria shows that the loss of this DAP-DAP bridge, inferring that there is no alternative L,D-transpeptidase responsible for this bridge in the intracellular niche. Importantly, the DAP-DAP bridge is present in all atypical crosslinked muropeptides (ISM-1, ISM-2, ISM-4) (**new Fig. 5**). The new immune response assays performed in cultured fibroblasts with this Δ*ycbB*Δ*ynhG* double mutant showed that the lack of this DAP-DAP bridge does not affect the capacity of intracellular S. Typhimurium to attenuate NF-κB nuclear translocation (**new Fig. 6C**). We however show that this mutant is still able to modify terminal D-alanine to alaninol (**S6 Fig**) and that this modification is sufficient to attenuate NF-κB nuclear translocation (**Fig. 7**). These new data led us to assign a "protective" role of these atypical muropeptides in envelope homeostasis as intracellular *S.* Typhimurium adapts to the harsh intra-phagosomal niche. An evidence for perturbation in the envelope of intracellular bacteria is the massive release of outer membrane vesicles from intra-phagosomal S. Typhimurium (García-del Portillo et al., Infect. Immun. 1997, PMID: 8975888)
Regarding the effect of a deficiency in the D,L-endopeptidase EcgA, we generated a double mutant Δ*ecgA*Δ*nlpC*, lacking the two enzymes known to cleave the D-Glu(γ)-*m*-DAP bond. These enzymes should be responsible for generating the atypical ISM-1 muropeptide in the PG of intracellular bacteria (**see Fig. 2**). We infected BJ5-ta human fibroblasts with this double mutant and were unable to detect in the PG of intracellular bacteria. However, the relative abundance of ISM-1 is extremely low (0.06%), so we are uncertain on whether this atypical ISM-1 is still present in the PG of Δ*ecgA*Δ*nlpC.* We nevertheless performed infection assays and analyzed NF-κB nuclear translocation in reporter fibroblasts. No major differences were seen compared to wild type, so we conclude more work it is needed to assess which are the responsible enzymes for generating the ISM-1 muropeptide. We plan in future work to overexpress these two enzymes, EcgA and NlpC, in artificial media mimicking the phagosomal conditions and isolate PG to discern whether the relative amounts of ISM-1 increase.

- In the methods it states “For some experiments requiring large quantities of peptidoglycan from intracellular bacteria, fibroblasts were infected with the phoP7953::Tn10 mutant, which overgrow inside this host cell type [44]. Previous control assays showed no differences in the muropeptide pattern of wild type and phoP7953::Tn10 growing extracellularly or inside fibroblasts.
  - Please, indicate exactly throughout the manuscript, for which experiments the strain was used. This information cannot be found anywhere in the manuscript.
  - It is mentioned that previous experiments didn’t show any differences to WT bacteria. Please show these data or cite the original work where they are published.

- This *phoP7953*::Tn*10* mutant, which proliferates at high rate inside fibroblasts (Cano et al. Infect. Immun. 2001, PMID: 11553591) was used exclusively to collect enough amount of the muropeptides corresponding to the peaks 14 and 16 shown in **Figure S5**. To obtain enough material was fundamental **to perform the subsequent mass spectrometry analyses of peaks 14 and 16 collected from the chromatographic column**. Note that the HPLC chromatograms shown in this figure correspond to the analysis performed with material isolated from intracellular **wild type bacteria**. These muropeptides, which correspond to the ISM muropeptides M3-alaninol and D33-alaninol (M3-X, D33-X), respectively, appear with similar abundance in both wild type and *phoP7953*::Tn*10* strains.

Reviewer #2: 1) The authors compare the PGN composition of in vitro vs in cellulo (Fibroblast) grown Salmonella. The comparative analysis shows several changes in the PGN composition such as an increase of L,D muropeptides (Table 1) while overall, the degree of crosslinking remained stable. Also, the authors show a decrease in anhydromuropeptides which indicates an increase in lytic transglycosylase activity in cellulo. The authors also describe the presence of novel muropeptides reflecting the trimming of a dimer by an amidase, a L,D-peptidase or a D,L-peptidase. Finally, the authors identify an unknown modification specific to in cellulo grown bacteria and propose that the modification is due to an alaninol residue at position 4 of stem peptide replacement the canonical D-Ala. The data is overall convincing but lacks evidence of reproducibility. For example, in none of the relevant tables (Table 1 and Table S2) are their experimental errors included. How often was the data of Figure 1A obtained to ensure that the ISMs are truly specific? Also, the best in vitro control would have been to have systematically Salmonella grown in DMEN instead of LB as this is the media to which Salmonella were exposed in cellulo (missing in the main figures and only tested in FigS5).

Please, see response relative to the lack of credits to our previous work regarding the protocol used to purify PG from intracellular *Salmonella*. Based on the comments of this reviewer, our interpretation is that our protocol of preparing peptidoglycan from intracellular has not been properly understood. We recall that samples of peptidoglycan from intracellular bacteria derive from multiple independent biological replicates(minimum, *n* = 10), **being necessary to pool the minute amounts of intracellular bacteria that is obtained BEFORE the step of boiling in SDS** to further process the samples for peptidoglycan purification.

Regarding the control in DMEM, we included it in the HPLC analysis. This sample did not show any trace of the unique muropeptides (ISM) seen in intracellular bacteria, even

for the most abundant (ISM 3,5,6) that were detected as the peaks 14 and 16 shown in Fig. S5. For this reason, we see unlikely that analyzing this sample by untargeted MS/MS we can obtain evidence of changes seen in intracellular bacteria were already in the PG of bacteria grown in DMEM. If so, they should have been detected by HPLC.

2) The authors present data in Fig1 and FigS5 that have been acquired by different HPLC-MS systems. In Fig1, table 1 and S2, the authors acquired data from uHPLC QTOF and extract total ion counts. In FigS5, it is based on a nanoLC system coupled to UV absorbance and Triple TOF. How comparable are these two analyses? Do the authors get the same percentage of muropeptides using these two quantification technics? How reliable if the TIC quantification given the fact that each muropeptide might have a different efficiency of ionization.

In our opinion, the fact that both techniques converged in the discovery of the same new muropeptides (ISM 3 and 5 in untargeted MS/MS that matched peaks 14 and 16 in the HPLC MS system) supports a strong correspondence in the results.

3) Technically, this analysis is a challenge. I was wondering why the authors lyse first the fibroblast before pursuing the SDS boiling and PGN purification. SDS boiling is used to solubilize completely the bacterial cell and inactivated any potential PGN hydrolytic activity. Since the authors suggest there is muropeptide trimming, how much of it is a technical artifact due to the prior lysis of the fibroblast? Why haven't the authors directly boiled the bacteria-fibroblast tandem? PGN is insoluble so I do not see the reason to perform a two-step protocol. Is it because despite the use of SDS, there will be too much of cellular debris contaminating the PGN preparation? Since the authors claim that bacterial enzymes are likely involved in this trimming of PGN, it would have been interesting to have the PGN composition of the corresponding mutants KO. Alternatively, it could be due to host enzymes. It would have been a plus to recover the fibroblast protein content after lysis
and incubate it with PGN from in vitro grown Salmonella and analyze the composition after incubation.

The reviewer is referred to the careful description of our protocol of isolation of PG provided in lines 389-398 of the manuscript. **Note that using a single flask it is not possible to get enough PG by high-speed centrifugation (estimate of ~10e7-10e8 intracellular bacteria).** Because of this, a minimum of 140 flasks were infected and the bacterial pellets further pooled for SDS boiling. This is tremendous work even for a single strain.

4) Then, the authors claim that the changes in PGN composition, in particular, the novel muropeptides carrying potentially alaninol are involved in dampening the immune response. The authors synthesize the corresponding stem peptides as Nod1 sensing does not require the sugar backbone for sensing. The authors show that the stem peptide carrying 2-amino-propan-1-ol is poorly sensed by NF-kB reporter cells expressing a p65-GFP. I assume that this is Nod1 mediated but not shown. A Nod1 KO is missing. Then, although the images seem convincing, I would like to see a really quantification of micro-injected cells that have p65-GFP in the nucleus (and not inject as some of the nucleus positive cells do not seem to have been injected). From the images presented the stem peptide carrying 1-amino-propa-2-ol also seems affected. The authors conclude from their data that this modification is a potential mechanism of escape to the immune system. I disagree. The muropeptides in question are of low abundance and the Nod1 ligand M4 is actually increased in cellulo (Table S2). Thus, if anything the potential to activate Nod1 is higher in intracellular Salmonella.

Furthermore, if we believe the in cellulo muropeptide composition data, it suggests an increase in lytic transglycosylase activity which is prone to release soluble muropeptides. So, if anything can be inferred from the data is that intracellular Salmonella have a heightened potential for activating Nod1.

Quantification of percentage of microinjected cells showing nuclear translocation of GFP-p65 is easy. This information is now provided in the new submitted version. Regarding the mechanism of Nod1 activation in fibroblasts persistently infected with *Salmonella*, **which was not the scope of the study**, the author is referred to our publication with whole bacteria in which we demonstrated that intracellular *Salmonella* attenuates the NF-κB response **in both mouse and human fibroblasts**:
Single-cell analyses reveal an attenuated NF-κB response in the Salmonella-infected fibroblast.
Ramos-Marquès E, Zambrano S, Tiérrez A, Bianchi ME, Agresti A, García-Del Portillo F. Virulence. 2017 Aug 18;8(6):719-740. doi: 10.1080/21505594.2016.1229727. Epub 2016 Aug 30. PMID: 27575017

Experiments in Nod1 KO cells were not performed for two reasons. First, to our knowledge, there is no Nod1 KO mouse cell line with a knock-in reporter to monitor NF-κB-mediated responses. Second, the manuscript describes a detailed structural analysis of the PG isolated from intracellular (both wild type and a large series of mutants) and it was focused on testing effects on the modifications found in the master pro-inflammatory regulator NF-κB. We consider dissecting the mechanism(s) of attenuation translates in adding much more information and requires a plenty of additional assays that were the initial scope of this work. Logically, we are interested in such mechanisms and are currently initiating in this line, involving assays directed to analyze fate of intracellular bacteria in the presence/absence of Nod1/Nod2 using cells from the respective KO mice as well as drugs that inhibit specifically these receptors.

Related to the previous point, the authors claim page 14, line 281-282 that contrary to data of the literature, the tetra peptide is a stimulatory molecule. That is wrong. M4 is a poor agonist of the human Nod1 (reference 37) but the best agonist of the mouse Nod1, which is the one being tested in this work (PMID: 16211083). In fact, this distinction between ligand specificity between human and mouse Nod1 raises another question since the PGN composition was performed in human and rat fibroblast cell line but biological activity was tested in mouse fibroblast. Do the authors get the same results for their synthetic stem peptides in human cells? And is the PGN composition of intracellular Salmonella identical in mouse fibroblast?

The experiment in human cells cannot be done since there is no similar reporter human fibroblasts to test in situ effect of the microinjected peptides.

Regarding the composition of PG in intracellular *Salmonella* obtained from "mouse" cells it is pending. We prioritized rat and human cells given our long expertise using cell lines of these origins (NRK-49F and BJ5-ta). Given the high structural similarity found in the PG of non-proliferating intracellular *Salmonella* irrespective of these two animal origins, we however do not expect large differences in mouse cells. This is a tremendous amount of work (more including wild type and mutant strains), to test a third animal origin recovering non-proliferating intracellular *Salmonella*, and because of it, we gave priority to the synthetic peptides and the monitoring of NF-kB activity.

5) Even if the authors might want to publish elsewhere the synthesis of the tetrapeptides, they should have submitted a confidential description of the methods and validation of the structures for this reviewer to be able to appreciate their quality.

This information, which integrates all steps of the synthesis and the validation by RMN and mass spectrometry, was not included since it occupies nearly 20 pages. As requested, it is now included in Supplementary Information, with all the validation data supporting the purity of the different peptides.

Part III – Minor Issues:

Reviewer #1: •There are several typos and broken sentence structures throughout the manuscript. Please check again carefully. The following is only a selection:

- L71: "... when persists ..." there is a word missing
- L73: place OMV in brackets, check commas
- L108: "... phenomenon has also recently been ..." insert been
- L118: "... architecture of non-proliferative ..."
- L551: Please, name the other tagged protein (MltF, MltB or MepA?)

Corrected in the revised version.

Reviewer #2: Page 6, line 118 add "of" after "PG architecture"
Page 6, line 120 and everywhere else in the manuscript. Use uHPLC and HPLC which mean ultra high-pressure liquid chromatography, and high-pressure liquid chromatography. Please, avoid using the term ultra sensitive liquid chromatography or UPLC. Technical terms should be precise even if that means repeating them over and over. Figure S5 is missing the F label on the panel F.

Corrected in the revised version.
